# Mutant THAP11 causes cerebellar neurodegeneration and triggers TREM2-mediated microglial activation in mice

**DOI:** 10.1172/JCI178349

**Published:** 2025-06-03

**Authors:** Eshu Ruan, Jingpan Lin, Zhao Chen, Qianai Sheng, Laiqiang Chen, Jiating He, Xuezhi Duan, Yiyang Qin, Tingting Xing, Sitong Yang, Mingtian Pan, Xiangyu Guo, Peng Yin, Xiao-Jiang Li, Hong Jiang, Shihua Li, Su Yang

**Affiliations:** 1 State Key Laboratory of Bioactive Molecules and Druggability Assessment, Guangdong Provincial Key Laboratory of Non-human Primate Research, Guangdong-Hong Kong-Macau Institute of CNS Regeneration, and; 2 Guangdong Basic Research Center of Excellence for Natural Bioactive Molecules and Discovery of Innovative Drugs, Jinan University, Guangzhou, China.; 3 Department of Neurology, Xiangya Hospital,; 4 Key Laboratory of Hunan Province in Neurodegenerative Disorders, and; 5 National Clinical Research Center for Geriatric Disorders, Xiangya Hospital, Central South University, Changsha, China.; 6 Department of Neurology, The Third Xiangya Hospital of Central South University, Changsha, China.; 7 Furong Laboratory and; 8 Brain Research Center, Central South University, Changsha, China.

**Keywords:** Genetics, Neuroscience, Mouse models, Neurodegeneration, Protein misfolding

## Abstract

Abnormal expansions of the CAG trinucleotide repeat within specific gene exons give rise to polyglutamine (polyQ) diseases, a family of inherited disorders characterized by late-onset neurodegeneration. Recently, a new type of polyQ disease was identified and named spinocerebellar ataxia 51 (SCA51). SCA51 is caused by polyQ expansion in THAP domain containing 11 (THAP11), an essential transcription factor for brain development. The pathogenesis of SCA51, particularly how mutant THAP11 with polyQ expansion contributes to neuropathology, remains elusive. Our study of mouse and monkey brains revealed that THAP11 expression is subject to developmental regulation, showing enrichment in the cerebellum. However, knocking down endogenous THAP11 in adult mice did not affect neuronal survival. In contrast, expressing mutant THAP11 with polyQ expansion led to pronounced protein aggregation, cerebellar neurodegeneration, and motor deficits, indicating that gain-of-function mechanisms are central to SCA51 pathogenesis. We discovered activated microglia expressing triggering receptor expressed on myeloid cells 2 (TREM2) in the cerebellum of a newly developed SCA51 knockin mouse model. Mechanistically, mutant THAP11 enhanced the transcription of *TREM2*, leading to its upregulation. The loss of TREM2 or depletion of microglia mitigated neurodegeneration induced by mutant THAP11. Our study offers the first mechanistic insights to our knowledge into the pathogenesis of SCA51, highlighting the role of TREM2-mediated microglial activation in SCA51 neuropathology.

## Abstract 2

Mutant THAP11 with polyglutamine repeat expansion induces TREM2 upregulation and microglial activation, which contributes to cerebellar degeneration in mouse models of SCA51.

## Graphical abstract

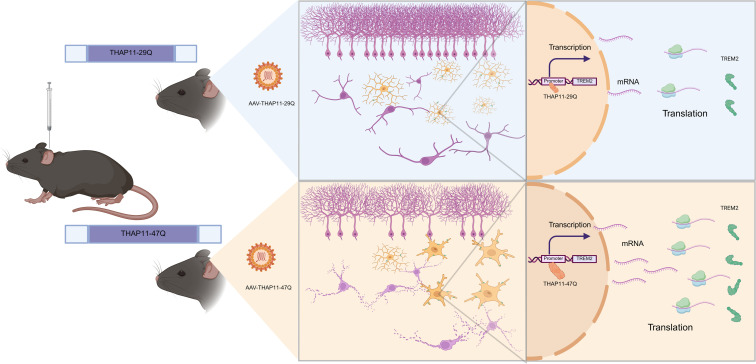

## Introduction

The expanded CAG trinucleotide repeat in the coding region of selected genes is a common theme in the polyglutamine (polyQ) family of neurodegenerative diseases. For over 2 decades, it has been recognized that polyQ diseases encompass 9 inherited disorders, including Huntington’s disease, spinal bulbar muscular atrophy, dentatorubral-pallidoluysian atrophy, and spinocerebellar ataxia (SCA) types 1, 2, 3, 6, 7, and 17 (1, 2). In each of these polyQ diseases, the gene carrying the expanded CAG trinucleotide repeat translates into proteins with an elongated polyQ tract, which are prone to aggregate and cause brain region–specific neurodegeneration (3, 4). The monogenic nature of polyQ diseases facilitates the generation of various disease models to investigate the underlying pathological mechanisms. While polyQ diseases are primarily caused by toxic gain-of-function effects, partial loss of endogenous protein functions may also contribute to disease pathogenesis (5–7).

In 2023, a new polyQ disease was identified and named SCA type 51 (SCA51) (8). The expansion of the CAG trinucleotide repeat was discovered in exon 1 of the THAP domain containing 11 (*THAP11*) gene in 2 distinct families in China and 1 patient in the United Kingdom. The number of CAG repeats ranges from 20 to 38 in healthy individuals but increases to above 45 in SCA51 patients. The characteristic symptoms of SCA51 patients include gait ataxia and dysarthria. MRI revealed obvious cerebellar atrophy (8, 9). *THAP11*, alternately named *RONIN*, encodes a transcription factor that belongs to an evolutionarily conserved protein family carrying specific zinc finger motifs termed THAP (10, 11). Emerging evidence indicates that THAP11 is essential for brain development, as *Thap11* deletion or mutation causes embryonic lethality or brain malformations in mice and zebrafish (12–14). Given that SCA51 is a newly identified polyQ disease, there is currently a lack of animal models, and the pathogenic mechanisms resulting from the polyQ expansion in THAP11 remain elusive.

In the present study, we examined the expression of THAP11 in mouse and monkey brains. We found that THAP11 is developmentally regulated and specifically enriched in the cerebellum, which aligns with the cerebellum-specific symptoms observed in SCA51 patients. Knocking down endogenous THAP11 via CRISPR/Cas9-mediated genome editing did not cause obvious neuronal damage in the mouse brain. Conversely, expressing mutant THAP11 with polyQ expansion derived from one SCA51 patient led to the formation of inclusion bodies, accompanied by cerebellar neurodegeneration and impaired motor functions in mice. Through transcriptomic analysis, we showed that mutant THAP11 with polyQ expansion elicits microglial activation. These activated microglia have increased expression of triggering receptor expressed on myeloid cells 2 (TREM2), which is a microglia-specific protein and central regulator of microglial functions (15, 16). These findings were validated in a newly generated knockin (KI) mouse model expressing human mutant THAP11. Mechanistically, mutant THAP11 functioned as a transcriptional activator to upregulate TREM2 expression. In *Trem2* knockout (KO) mice and WT mice with microglial depletion, the neurodegeneration caused by mutant THAP11 was attenuated. Together, our findings demonstrate that TREM2-mediated microglial activation by mutant THAP11 is one important gain-of-function mechanism underlying SCA51 pathogenesis.

## Results

### The expression of THAP11 is developmentally regulated and enriched in the cerebellum.

**Figure 1:**
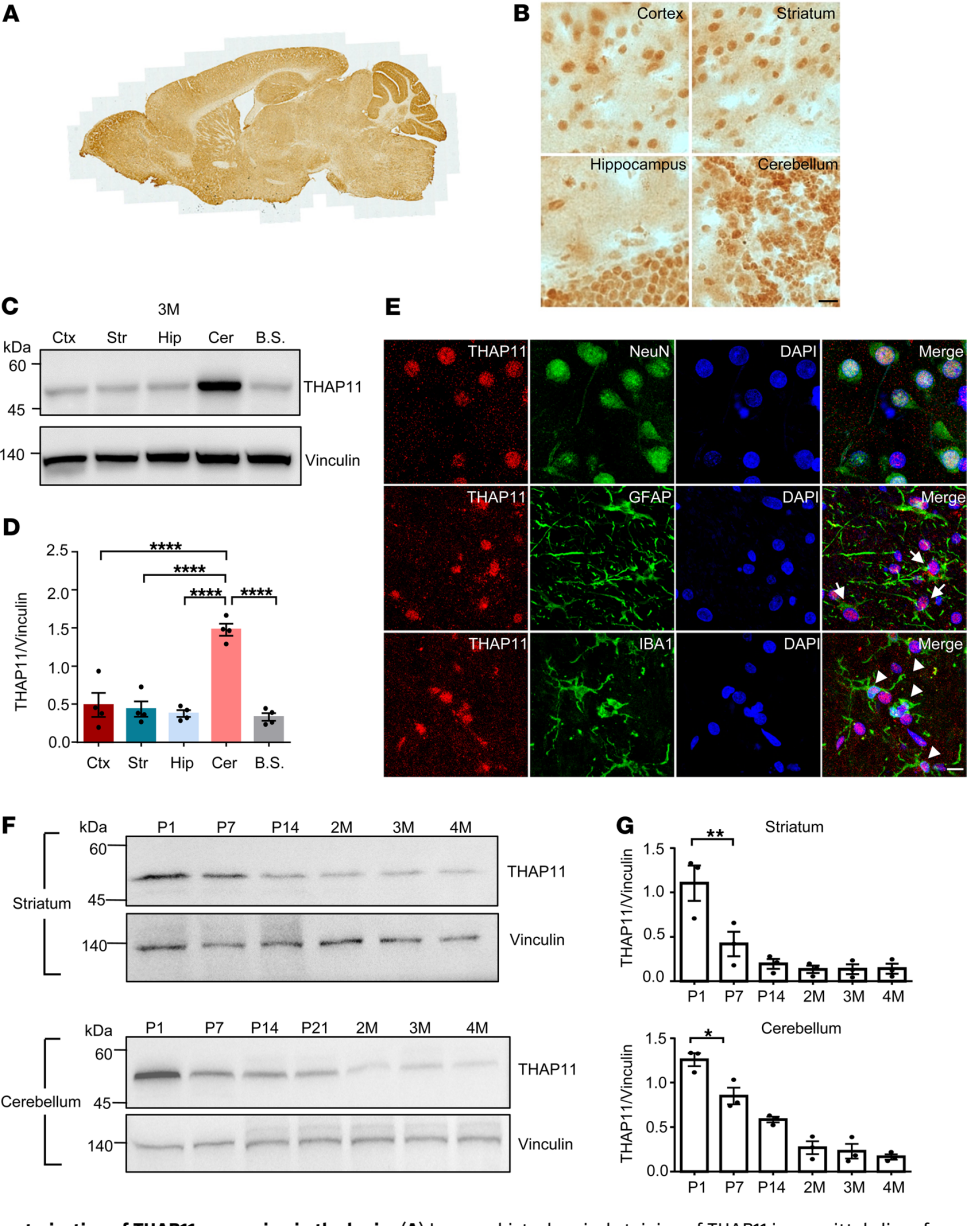
Characterization of THAP11 expression in the brain. (**A**) Immunohistochemical staining of THAP11 in a sagittal slice of mouse brain. (**B**) High-magnification (original magnification, ×40) images showing the expression of THAP11 in the cortex, striatum, hippocampus, and cerebellum (scale bar: 20 μm). (**C**) Western blotting analysis of THAP11 in different brain regions of 3-month-old mice (Ctx, cortex; Str, striatum; Hip, hippocampus; Cer, cerebellum; B.S., brain stem). Vinculin served as a loading control. (**D**) Quantification of Western blotting results in **C** (*n* = 4, 1-way ANOVA with Tukey’s post tests). (**E**) Double immunostaining of THAP11 with NeuN, GFAP, or IBA1 in the brain slices (arrows indicate astrocytes with THAP11 staining; arrowheads indicate microglia with THAP11 staining; scale bar: 10 μm). (**F**) Western blotting analysis of THAP11 expression in the striatum and cerebellum of differently aged mice (P1, P7, P14, and P21 indicate postnatal days 1, 7, 14, and 21; 2M, 3M, and 4M indicate 2, 3, and 4 months old). (**G**) Quantification of Western blotting results in **F** (*n* = 3, 1-way ANOVA with Tukey’s post tests). *P < 0.05; **P < 0.01; ****P < 0.0001. Data are presented as mean values ± SEM.

First, we characterized the expression of THAP11 in the mouse brain. The specificity of the THAP11 antibody was verified by Western blotting analysis using the murine N2a cells transfected with *Thap11* siRNAs (Supplemental Figure 1A; supplemental material available online with this article; https://doi.org/10.1172/JCI178349DS1). Immunohistochemistry results showed that THAP11 was widely expressed in various brain regions of adult mice, but was particularly enriched in the cerebellum, the brain region most affected in SCA51 (Figure 1, A and B). In both 1- and 3-month-old mice, Western blotting analysis revealed that the cerebellum exhibited the highest THAP11 expression, whereas other brain regions, including the cortex, striatum, hippocampus, and brain stem, had comparable expression levels (Figure 1, C and D and Supplemental Figure 1, B and C). We also examined the expression of THAP11 in different brain regions of 5-year-old cynomolgus monkeys (*Macaca fascicularis*) and found a similar pattern (Supplemental Figure 1, D and E). Using double immunofluorescence staining, we confirmed that THAP11 was ubiquitously expressed in the nuclei of neurons, astrocytes, and microglia (Figure 1E and Supplemental Figure 2, A and B). Within the cerebellum, abundant THAP11 expression was detected in the Purkinje cells and granule cells (Supplemental Figure 2C).

Given that most brain regions have similar levels of THAP11, we selected the striatum as a comparison to the cerebellum for the subsequent studies. As THAP11 is essential for development (12), we examined the expression of THAP11 in the striatum and cerebellum of mice at different postnatal ages, from postnatal day 1 (P1) to 4 months old. In both brain regions, the expression of THAP11 gradually declined from P1 to 2 months of age but remained detectable as the mice matured into adulthood (Figure 1, F and G). Similarly, we found an age-related decrease of THAP11 in the monkey cerebellum (Supplemental Figure 2, D and E). These results suggest that THAP11 is important for early brain development, and some of its functions could be retained in the adult brain.

### Loss of endogenous THAP11 does not affect neuronal survival in mice.

**Figure 2:**
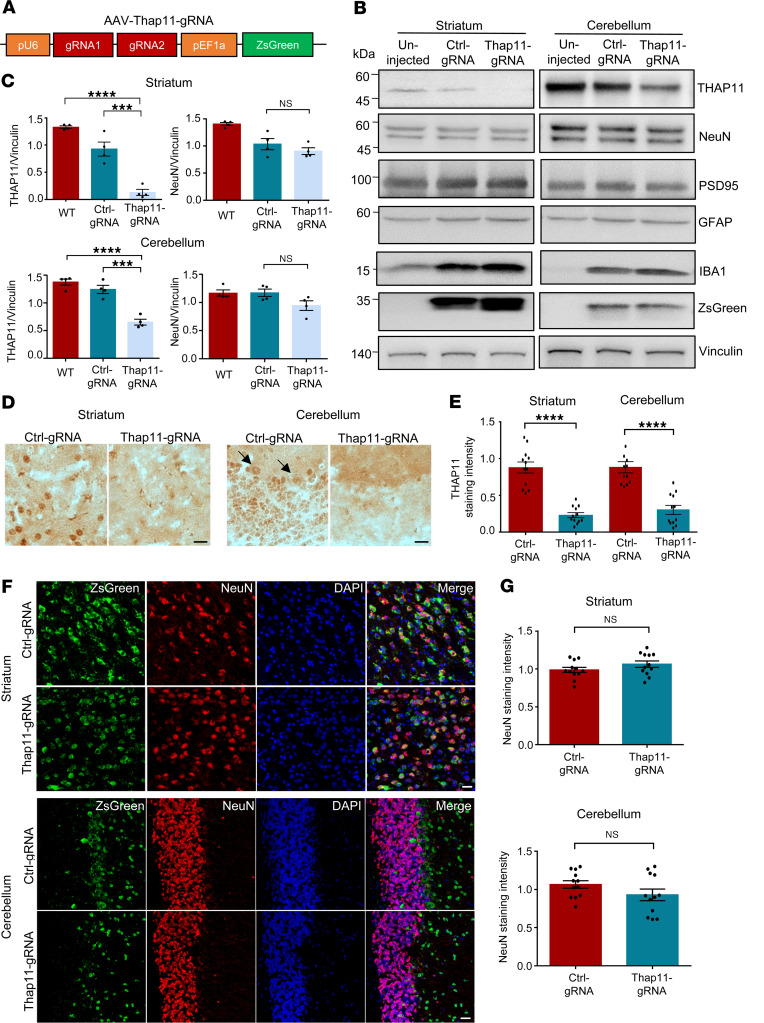
Knockdown of THAP11 does not affect neuronal survival in adult mice. (**A**) A schematic diagram of the AAV-Thap11-gRNA plasmid. (**B**) The germline Cas9 mice were either uninjected or injected with AAV-Ctrl-gRNA or AAV-Thap11-gRNA. Western blotting analysis was performed on THAP11, NeuN, PSD95, GFAP, and IBA1 in the striatum and cerebellum of these mice. ZsGreen was used to indicate AAV infection, and Vinculin served as a loading control. (**C**) Quantification of Western blotting results in **B** (*n* = 4, 1-way ANOVA with Tukey’s post tests). (**D**) Immunohistochemical staining showed the loss of THAP11 staining in the striatum and cerebellum area expressing AAV-Thap11-gRNA (arrows indicate Purkinje cells; scale bars: 20 μm). (**E**) Quantification of THAP11 staining intensity in **D** (*n* = 12 from 3 mice; 2-tailed Student’s *t* test). (**F**) Immunofluorescence staining of NeuN in the striatum and cerebellum of germline Cas9 mice injected with AAV-Ctrl-gRNA or AAV-Thap11-gRNA. ZsGreen fluorescence reflected the expression of gRNAs (scale bars: 20 μm). (**G**) Quantification of NeuN staining intensity in **F** (*n* = 12 from 3 mice, 2-tailed Student’s *t* test). ***P < 0.001, ****P < 0.0001. Data are presented as mean values ± SEM.

We wondered if a loss of endogenous THAP11 function could contribute to SCA51 pathogenesis. To that end, we utilized the CRISPR/Cas9 genome editing tools to knock down WT *Thap11* in mice. We designed 2 guide RNA (gRNA) sequences targeting exon 1 of *Thap11* and constructed adeno-associated virus (AAV) plasmids expressing these gRNAs (AAV-Thap11-gRNA) (Figure 2A). The genome editing efficiency of the designed gRNAs was verified by a T7E1 assay in the murine N2a cells transfected with the CRISPR/Cas9 plasmids (Supplemental Figure 3A). We also performed Western blotting to confirm the reduction of THAP11 protein (Supplemental Figure 3B).

We packaged the Thap11-gRNA plasmids into AAVs and delivered the AAVs into the striatum and cerebellum of 3-month-old germline Cas9 mice via stereotaxic injection. The germline Cas9 mice ubiquitously express the Cas9 nuclease (17), so genome editing should occur in the cells expressing AAV-Thap11-gRNA. One month later, the mice were sacrificed, and their brain tissues were collected for analyses. In both striatum and cerebellum, we noticed a significant reduction in THAP11 (Figure 2, B and C). However, the expression of neuronal marker proteins, including NeuN and PSD95, was not significantly changed (Figure 2, B and C and Supplemental Figure 3C), indicating that THAP11 knockdown did not affect adult neuronal survival. We also examined the reactive astrocyte marker GFAP and the microglia marker IBA1. Both proteins showed increased expression due to AAV injection, but no differences were found between tissues injected with control gRNA (AAV-Ctrl-gRNA) or AAV-Thap11-gRNA (Figure 2, B and C and Supplemental Figure 3C). We validated the reduction of endogenous THAP11 via immunohistochemistry and immunofluorescence staining (Figure 2, D and E and Supplemental Figure 3D). Nissl staining showed that the number of Purkinje cells was not changed in the cerebellum injected with AAV-Thap11-47Q (Supplemental Figure 3, E and F). Additionally, in the areas expressing zsGreen that reflected gRNA expression, we did not see significant changes in NeuN, IBA1, or GFAP staining intensity (Figure 2, F and G and Supplemental Figures 4 and 5). Together, these results indicate that the reduction of endogenous THAP11 does not negatively impact the survival of neurons in adult mice.

### The polyQ-expanded mutant THAP11 causes cerebellar neurodegeneration.

We switched our focus to the gain-of-function mechanisms. We amplified full-length human *THAP11* cDNAs carrying 29 or 47 CAG repeats from human HEK293 cells and one SCA51 patient. The cDNAs were used to construct PRK plasmids expressing WT THAP11 (THAP11-29Q) or mutant THAP11 with polyQ expansion (THAP11-47Q). HEK293 cells have been extensively utilized for expressing polyQ proteins and assessing their aggregation tendencies (18–21). We transfected these plasmids into HEK293 cells and detected robust protein expression by Western blotting. Both THAP11-29Q and THAP11-47Q formed oligomers in the transfected cells, but only THAP11-47Q formed aggregates, which can be recognized by the THAP11 antibody or the 1C2 antibody that targets the polyQ region (Supplemental Figure 6A). The THAP11-47Q aggregates were also identified by immunostaining in the transfected cells, which were predominantly localized in the nucleus (Supplemental Figure 6B). By MTT assay, we found that the viability of THAP11-47Q–expressing cells was significantly lower than THAP11-29Q–expressing cells (Supplemental Figure 6C), suggesting that mutant THAP-11 with polyQ expansion is cytotoxic.

**Figure 3:**
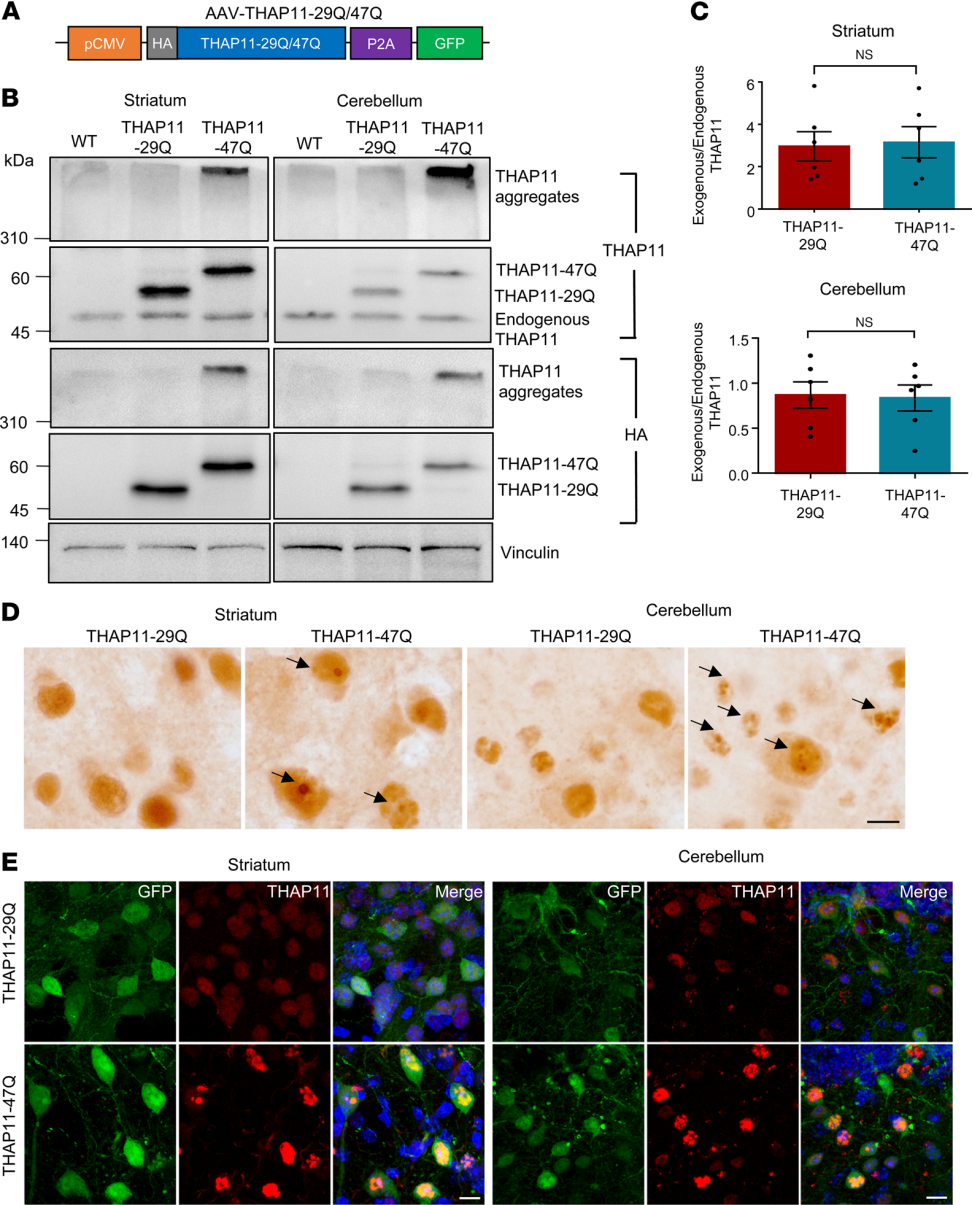
Mutant THAP11 with polyQ expansion forms aggregates in the mouse brain. (**A**) A schematic diagram of the AAV-THAP11-29Q and AAV-THAP11-47Q plasmids. (**B**) Western blotting analysis of THAP11 expression in the striatum and cerebellum of WT mice that were either uninjected or injected with AAV-THAP11-29Q or AAV-THAP11-47Q. THAP11 and HA antibodies were used to detect THAP11, and Vinculin served as a loading control. (**C**) The ratio of exogenous to endogenous THAP11 in the striatum and cerebellum of WT mice injected with AAV-THAP11-29Q or AAV-THAP11-47Q (*n* = 6, 2-tailed Student’s *t* test). (**D**) Immunohistochemical staining of THAP11 in the striatum and cerebellum of WT mice injected with AAV-THAP11-29Q or AAV-THAP11-47Q (arrows indicate THAP11-47Q aggregates; scale bar: 10 μm). (**E**) Immunofluorescence staining of THAP11 in the striatum and cerebellum of WT mice injected with AAV-THAP11-29Q or AAV-THAP11-47Q. GFP fluorescence reflected the AAV-infected cells (scale bars: 20 μm). Data are presented as mean values ± SEM.

Next, we constructed AAV plasmids expressing THAP11-29Q or THAP11-47Q with an N-terminal HA tag and a C-terminal P2A peptide linking GFP, driven by the ubiquitous CMV promoter (Figure 3A and Supplemental Figure 7A). The plasmids were packaged into AAVs (AAV-THAP11-29Q and AAT-THAP11-47Q). We delivered the AAVs to the striatum and cerebellum of 3-month-old WT mice via stereotaxic injection, with AAV-THAP11-29Q delivered to one hemisphere and AAV-THAP11-47Q delivered to the other hemisphere. One month after injection, the expression of THAP11-29Q and THAP11-47Q was confirmed using the THAP11 and HA antibodies, respectively (Figure 3B). THAP11-47Q formed extensive aggregates, which were detected in the stacking gel by Western blotting (Figure 3B) and by filter trap assays (Supplemental Figure 7, B and C). Importantly, the expression level of exogenous THAP11 was comparable to endogenous THAP11 in the cerebellum (Figure 3C). Histochemical staining revealed that the THAP11-47Q aggregates were localized in the nucleus. In the cerebellum, the THAP11-47Q aggregates were detected in both Purkinje cells and granule cells (Figure 3D). This result was further supported by double immunofluorescence staining, as THAP11-positive signal and GFP fluorescence overlapped in the injected striatum and cerebellum (Figure 3E). Comparison between the AAV-THAP11-29Q injected brain and the WT brain validated the specificity of the staining (Supplemental Figure 7D). We also injected AAV-THAP11-47Q into the cerebellum of 1-month-old WT mice and found less THAP11 aggregates 1 month after injection (Supplemental Figure 7, E and F), indicating a potential age-dependent decline in aggregate clearance.

**Figure 4:**
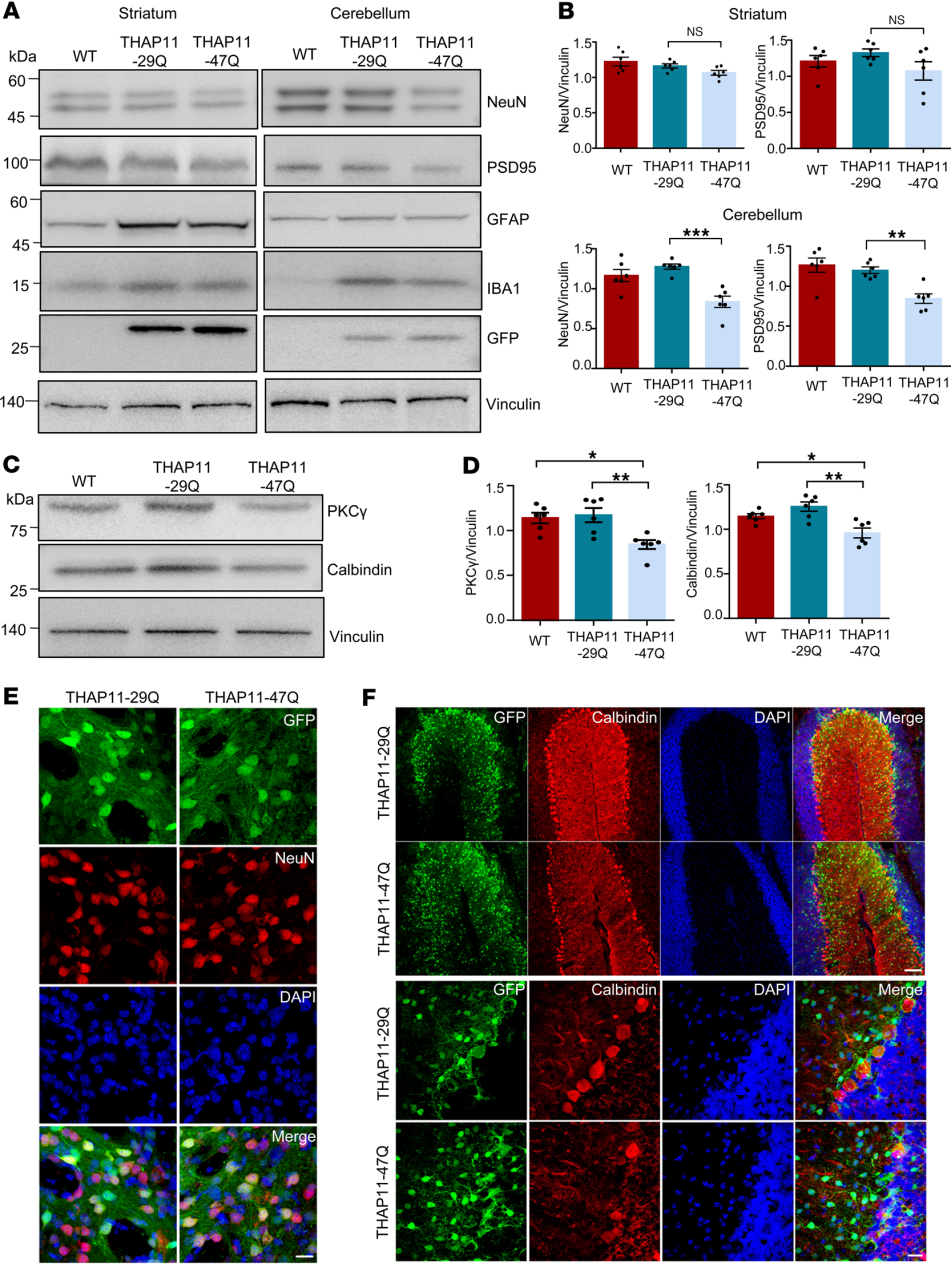
Mutant THAP11 with polyQ expansion causes cerebellar neuronal loss in mice. (**A**) Western blotting analysis of NeuN, PSD95, GFAP, and IBA1 expression in the striatum and cerebellum of WT mice that were either uninjected or injected with AAV-THAP11-29Q or AAV-THAP11-47Q. GFP indicated AAV infection, and Vinculin served as a loading control. (**B**) Quantification of Western blotting results in **A** (*n* = 6, 1-way ANOVA with Tukey’s post tests). (**C**) Western blotting analysis of PKCγ and calbindin expression in the cerebellum of WT mice that were either uninjected or injected with AAV-THAP11-29Q or AAV-THAP11-47Q. Vinculin served as a loading control. (**D**) Quantification of Western blotting results in **C** (*n* = 6, 1-way ANOVA with Tukey’s post tests). (**E**) Immunofluorescence staining of NeuN in the striatum of WT mice injected with AAV-THAP11-29Q or AAV-THAP11-47Q. GFP fluorescence reflected the AAV-infected cells (scale bar: 20 μm). (**F**) Immunofluorescence staining of calbindin in the cerebellum of WT mice injected with AAV-THAP11-29Q or AAV-THAP11-47Q. GFP fluorescence reflected the AAV-infected cells (scale bars: top, 100 μm; bottom, 20 μm). *P < 0.05, **P < 0.01, ***P < 0.001. Data are presented as mean values ± SEM.

We evaluated the level of neuronal damage in the striatum and cerebellum of the injected mice via Western blotting. In the striatum, the expression of THAP11-29Q or THAP11-47Q did not cause obvious changes in NeuN or PSD95, whereas in the cerebellum, the THAP11-47Q–expressing tissues showed a significant reduction of both proteins (Figure 4, A and B). The expression of GFAP and IBA1 was also induced by AAV injection, but no significant changes were found between THAP11-29Q– and THAP11-47Q–expressing tissues (Figure 4A and Supplemental Figure 8A). The Purkinje cells in the cerebellum are especially susceptible in several types of SCA (22–24). We examined the expression of 2 markers for Purkinje cells (calbindin and PKCγ) and found that both were significantly reduced in the presence of THAP11-47Q (Figure 4, C and D), suggesting that THAP11-47Q causes Purkinje cell degeneration. In agreement with the Western blotting results, immunofluorescence images showed that THAP11-47Q did not affect NeuN staining in the striatum but impaired the morphology and survival of Purkinje cells in the cerebellum, as demonstrated by a loss of calbindin staining (Figure 4, E and F). The neuronal morphology in the AAV-THAP11-29Q–injected brain closely resembled that of the WT brain, indicating that the overexpression of THAP11-29Q does not negatively affect neurons (Supplemental Figure 8, B and C). To further confirm the loss of Purkinje cells, we performed Nissl staining, which revealed that the number of Purkinje cells declined to 51% in THAP11-47Q–expressing cerebellum (Supplemental Figure 8, D and E). Therefore, the ectopic expression of THAP11-47Q causes Purkinje cell–specific degeneration.

### Mutant THAP11 expression in the cerebellum impairs the motor functions of mice.

**Figure 5:**
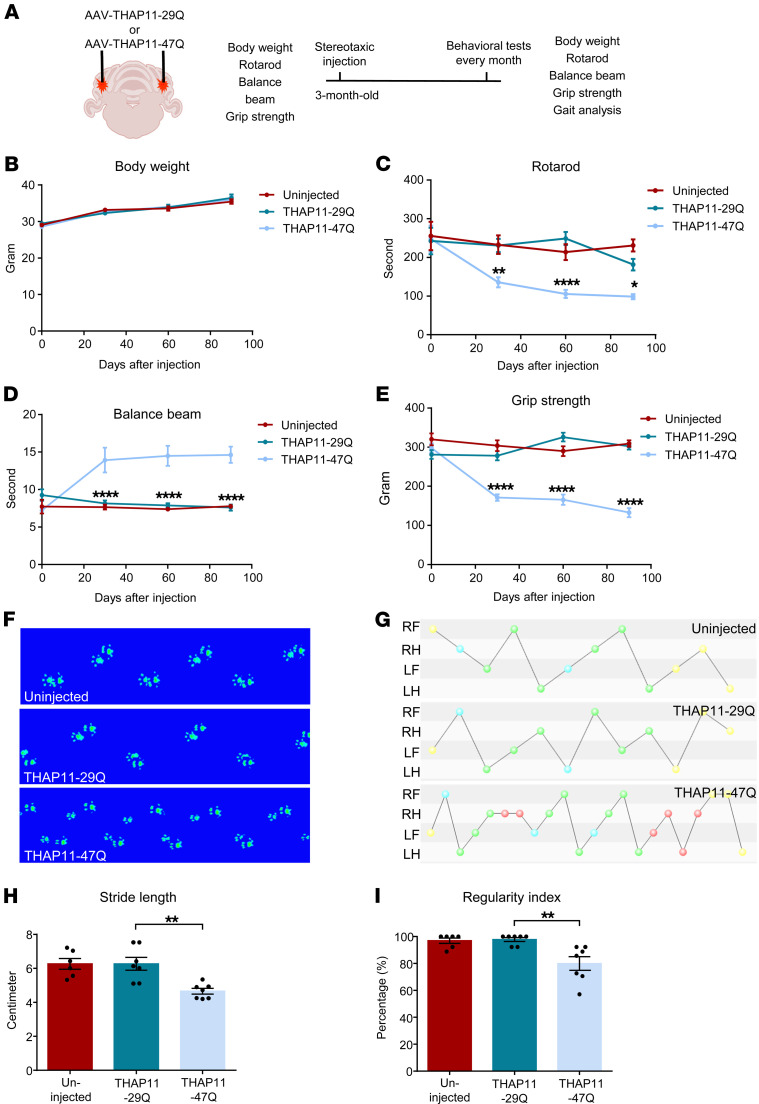
Mutant THAP11 with polyQ expansion causes motor deficits in mice. (**A**) A schematic diagram showing stereotaxic injection and the behavioral tests performed. (**B**–**E**) Three-month-old WT mice were either uninjected or injected with AAV-THAP11-29Q or AAV-THAP11-47Q in the cerebellum. The motor functions of these mice were assessed by body weight (**B**), rotarod (**C**), balance beam (**D**), and grip strength (**E**) before and monthly after injection. Mice injected with AAV-THAP11-47Q demonstrated significantly poorer performance in rotarod, balance beam, and grip strength tests (*n* = 6–7 per group, 2-way ANOVA tests). (**F** and **G**) Footprints (**F**) and step sequences (**G**) of the mice 30 days after injection were captured by the CatWalk system. For step sequences, the yellow dots were excluded automatically by the CatWalk analyzing software, the red dots indicate wrong sequences, the blue dots indicate the start of a pattern, and the green dots indicate parts of a pattern(abbreviations of the 4 paws: RF, right forward; RH, right hind; LF, left forward; LH, left hind). (**H** and **I**) The stride length (**H**) and regularity index (**I**) of the mice 30 days after injection were analyzed by the CatWalk system. The AAV-THAP11-47Q–injected mice had significantly smaller stride lengths and lower regularity index (*n* = 6–7 per group, 1-way ANOVA with Tukey’s post tests). *P < 0.05, **P < 0.01, ****P < 0.0001. Data are presented as mean values ± SEM.

To explore the potential effects of THAP11 expression on mouse behaviors, we bilaterally injected AAV-THAP11-29Q or AAV-THAP11-47Q into the cerebellum of 3-month-old WT mice. The motor functions of the mice before and after stereotaxic surgery were assessed by a battery of behavioral tests (Figure 5A). We did not see significant changes in the body weight of the mice (Figure 5B). Nonetheless, the mice injected with AAV-THAP11-47Q performed significantly worse in the rotarod test compared with the mice injected with AAV-THAP11-29Q (Figure 5C). In the balance beam test, the mice injected with AAV-THAP11-47Q showed poor coordination and spent more time walking through the beam (Figure 5D and Supplemental Video 1). These mice also had significantly weaker grip strength (Figure 5E). One typical symptom of SCA51 patients is ataxic gait. We utilized the CatWalk system to analyze the gait of the mice and found disrupted gait patterns in the mice injected with AAV-THAP11-47Q (Figure 5, F and G). These mice also had significantly smaller stride lengths and lower regularity index (Figure 5, H and I). Together, these results demonstrate that the expression of THAP11-47Q in the cerebellum leads to motor impairments in mice.

### The polyQ-expanded mutant THAP11 triggers microglial activation, characterized by TREM2 upregulation.

**Figure 6:**
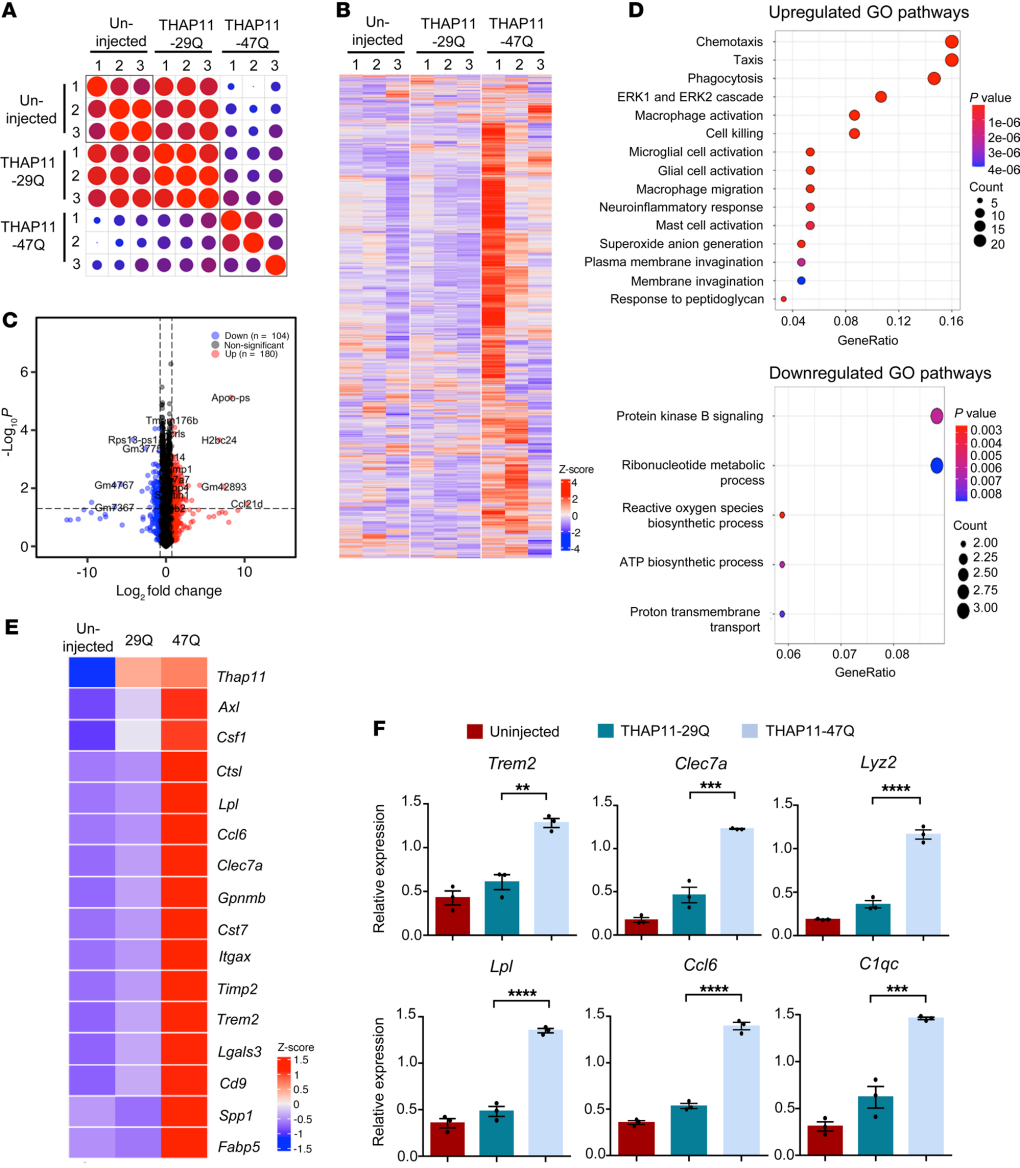
Transcriptome analysis reveals microglial activation in the presence of mutant THAP11 with polyQ expansion. (**A**) Correlation plot showing the overall similarities in the transcriptome among individual samples (WT uninjected and WT injected with AAV-THAP11-29Q or AAV-THAP11-47Q; 3 samples for each group). (**B**) Heatmap showing the overall gene transcription in WT mice that were either uninjected or injected with AAV-THAP11-29Q or AAV-THAP11-47Q. (**C**) Volcano plot illustrating upregulated or downregulated DEGs when comparing the cerebellum tissues injected with AAV-THAP11-29Q or AAV-THAP11-47Q. (**D**) GO enrichment analysis of the biological pathways that are upregulated or downregulated. (**E**) Heatmap showing the relative levels of marker genes for microglial activation in WT mice that were either uninjected or injected with AAV-THAP11-29Q or AAV-THAP11-47Q. The relative expression of *Thap11* was also included. (**F**) Quantitative real-time PCR analysis of selected genes verified the RNA-Seq results (*n* = 3, 1-way ANOVA with Tukey’s post tests). **P < 0.01, ***P < 0.001, ****P < 0.0001. Data are presented as mean values ± SEM.

As THAP11 is a transcription factor, we speculate that the polyQ expansion could alter the function of THAP11 in transcriptional regulation. To that end, we performed RNA sequencing (RNA-Seq) using cerebellum tissues from the mice that were either uninjected or injected with AAV-THAP11-29Q or AAV-THAP11-47Q. In terms of overall transcriptional profiles, the THAP11-29Q–expressing tissues closely resembled the uninjected tissues, whereas the THAP11-47Q–expressing tissues showed a divergent pattern (Figure 6, A and B). By comparing the THAP11-47Q–expressing tissues to THAP11-29Q–expressing tissues, we identified 284 differentially expressed genes (DEGs), with 180 upregulated and 104 downregulated genes (Figure 6C). Gene Ontology (GO) enrichment analysis of these DEGs revealed significantly upregulated pathways that include phagocytosis, ERK cascade, and cell killing as well as downregulated pathways that include PKB signaling and metabolic process (Figure 6D). Interestingly, we found that microglial activation markers constituted a significant portion of the identified DEGs, and their levels were upregulated in the THAP11-47Q–expressing tissues (Figure 6E). Using quantitative real-time PCR, we verified that selected DEGs were indeed significantly changed in the cerebellum injected with AAV-THAP11-47Q (Figure 6F), consistent with the RNA-Seq results. Among these DEGs, *Trem2* is a microglia-specific gene and a master regulator of microglia activation (25, 26).

**Figure 7:**
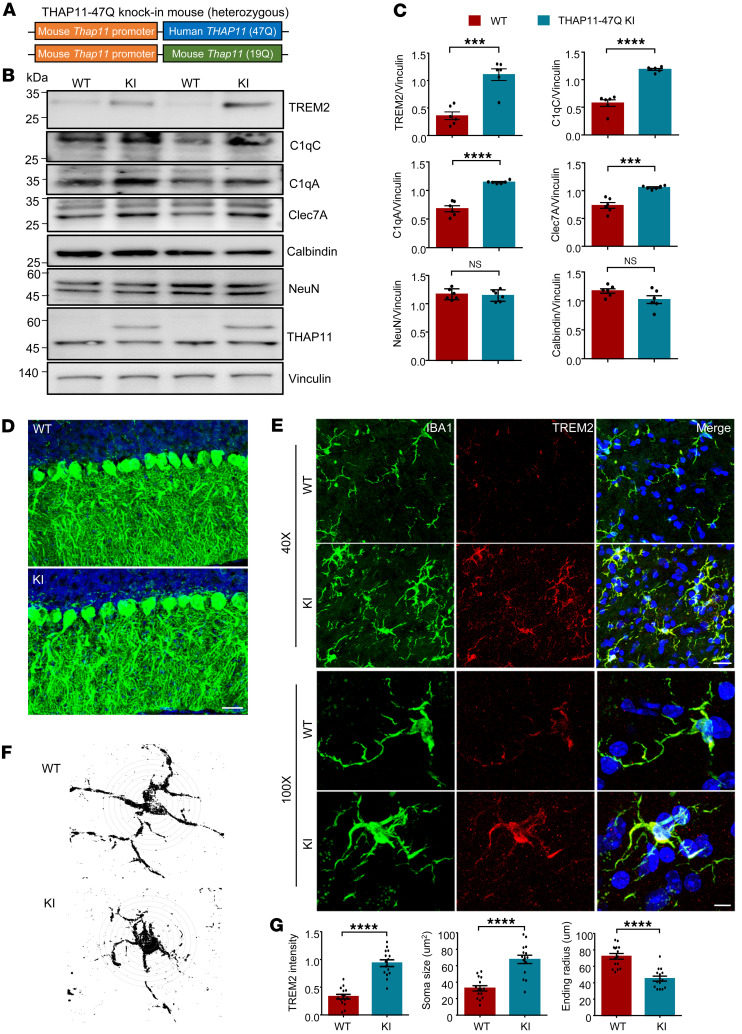
Microglial activation in THAP11-47Q KI mice. (**A**) A schematic diagram of the THAP11-47Q KI mouse model. (**B**) Western blotting analysis of NeuN, calbindin, TREM2, C1qA, C1qC, and Clec7A in the cerebellum of 7-month-old WT and THAP11-47Q KI mice. THAP11 antibody detected the expression of WT and polyQ-expanded THAP11. Vinculin served as a loading control. (**C**) Quantification of Western blotting results in **B** (*n* = 6, 2-tailed Student’s *t* test). (**D**) Immunofluorescence staining of calbindin in the cerebellum of 7-month-old WT and THAP11-47Q KI mice (scale bar: 20 μm). (**E**) Immunofluorescence staining of IBA1 and TREM2 in the cerebellum of 7-month-old WT and THAP11-47Q KI mice (scale bar: 40X, 50 μm; 100X, 10 μm). (**F**) Sholl analysis of microglia morphology in the cerebellum of 7-month-old WT and THAP11-47Q KI mice. (**G**) Quantification of TREM2 staining intensity, soma size, and ending radius of microglia (*n* = 15 from 3 mice, 2-tailed Student’s *t* test). ***P < 0.001, ****P < 0.0001. Data are presented as mean values ± SEM.

To validate the findings in a context that is more physiologically relevant, we generated a KI mouse model (THAP11-47Q KI) expressing human *THAP11* carrying 47 CAG repeats under the control of the endogenous mouse *Thap11* promoter (Figure 7A). Given the over 90% sequence homology between mouse and human THAP11, and based on previously published polyQ disease KI models (27–29), we conducted a comparative analysis between THAP11-47Q KI mice and WT mice to investigate the neuropathological effects associated with the presence of mutant THAP11. The cerebellum tissues from 7-month-old heterozygous THAP11-47Q KI mice were collected for Western blotting analysis. The expression of NeuN and calbindin remained unchanged (Figure 7, B and C), which is consistent with previous reports that intermediate polyQ expansion does not result in overt neurodegeneration in mice (30–32). In contrast, the expression of TREM2, C1qA, C1qC, and Clec7A was significantly upregulated (Figure 7, B and C), indicating that microglial activation precedes neuronal death in THAP11-47Q KI mice. At the transcriptional level, the microglial activation markers identified from RNA-Seq were also significantly increased in the cerebellum of THAP11-47Q KI mice (Supplemental Figure 9). Immunofluorescence staining revealed normal Purkinje cell morphology and increased TREM2 staining intensity in the cerebellum of 7-month-old THAP11-47Q KI mice (Figure 7, D and E). In addition, the microglia in THAP11-47Q KI mice were activated as the soma became larger and the ending radius of the process was shortened (Figure 7, F and G).

### TREM2 deletion ameliorated neurodegeneration caused by mutant THAP11 with polyQ expansion.

To verify if THAP11 functions in the microglia to regulate TREM2 expression, we transfected the human HMC3 microglial cells with THAP11-29Q or THAP11-47Q plasmids and examined TREM2 protein levels. HMC3 cells are known to endogenously express TREM2 (33, 34). We found a significant increase of TREM2 in the cells expressing THAP11-47Q, but not in the cells expressing GFP or THAP11-29Q (Supplemental Figure 10, A and B). In contrast, full-length Ataxin 3 (ATXN3-22Q or ATXN3-77Q) or Huntingtin exon 1 (HTT-Ex1-20Q or HTT-Ex1-150Q) did not change TREM2 expression in HMC3 cells (Supplemental Figure 10, A and B), indicating that TREM2 upregulation is specifically triggered by THAP11-47Q, but not other polyQ proteins. One previous study identified a conserved THAP11 binding motif in the gene promoter regions (35). We searched the JASPAR database (36) and found 2 similar sequences within the *TREM2* promoter (Supplemental Figure 11A). We performed a ChIP assay using HMC3 cells to examine the binding of THAP11 on the *TREM2* promoter. In the cells expressing THAP11-47Q, we precipitated a significantly higher amount of DNA corresponding to the *TREM2* promoter (Supplemental Figure 11, B and C), suggesting that THAP11-47Q has a stronger binding affinity to the *TREM2* promoter than THAP11-29Q. Furthermore, we constructed a luciferase reporter controlled by the *TREM2* promoter containing 1,500 bp upstream of the translation start site. In HMC3 cells, THAP11-47Q, but not THAP11-29Q, resulted in a significantly higher luciferase intensity (Supplemental Figure 11, D and E), indicating that while overexpression of WT THAP11 does not lead to *TREM2* upregulation, mutant THAP11 with polyQ expansion enhances *TREM2* transcription. This result is consistent with our RNA-Seq data showing that THAP11-29Q overexpression in the mouse cerebellum did not significantly increase *Trem2* mRNA levels.

**Figure 8:**
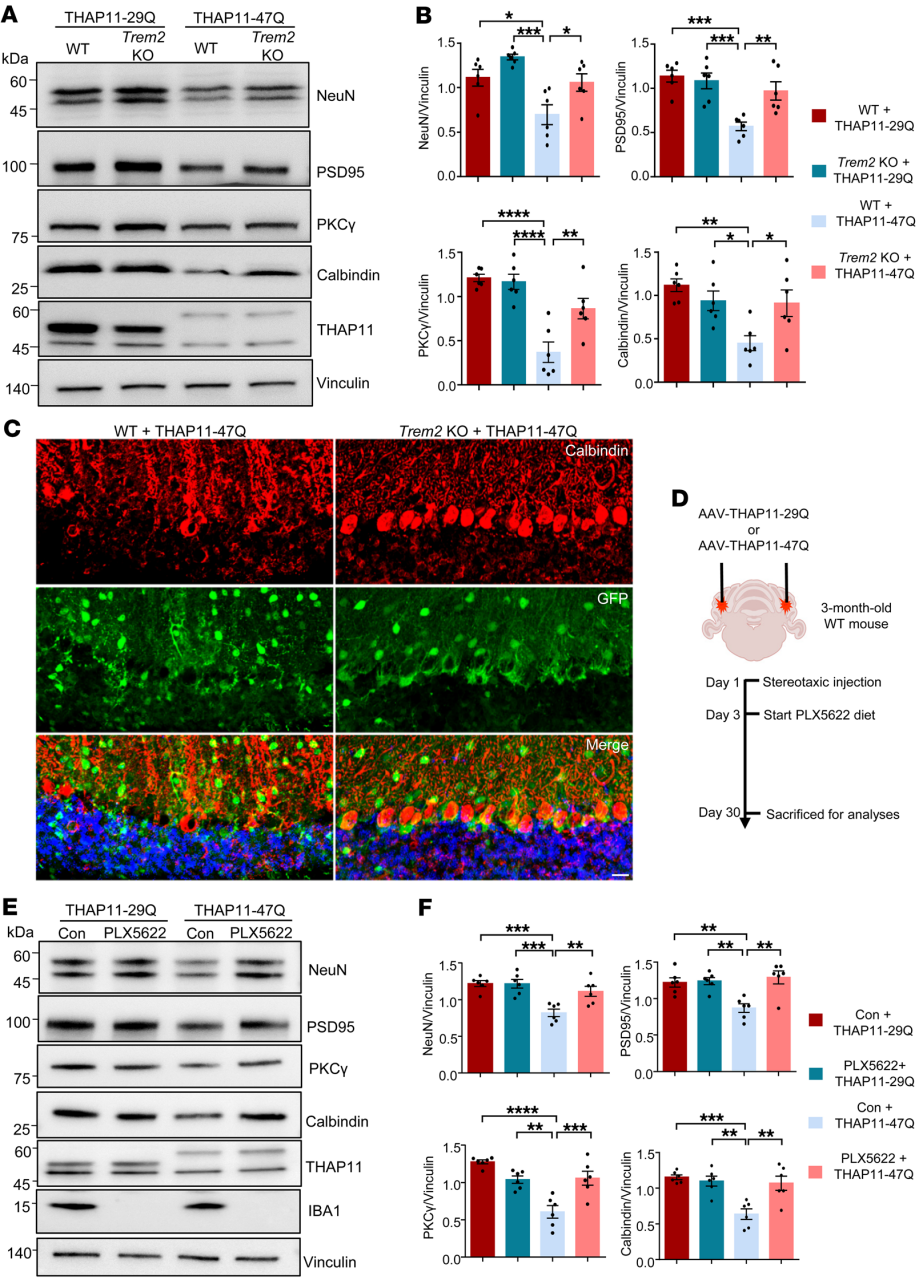
Loss of TREM2 attenuates mutant THAP11 neurotoxicity. (**A**) Western blotting analysis of NeuN, PSD95, PKCγ, and calbindin expression in the cerebellum of WT and *Trem2* KO mice injected with AAV-THAP11-29Q or AAV-THAP11-47Q. Vinculin served as a loading control. (**B**) Quantification of Western blotting results in **A** (*n* = 6, 1-way ANOVA with Tukey’s post tests). (**C**) Immunofluorescence staining of calbindin in the cerebellum of WT and *Trem2* KO mice injected with AAV-THAP11-47Q. GFP fluorescence reflected the AAV-infected cells (scale bar: 20 μm). (**D**) A schematic diagram showing stereotaxic injection and drug administration conducted on WT mice. (**E**) Western blotting analysis of NeuN, PSD95, PKCγ, calbindin, and IBA1 expression in the cerebellum of WT mice injected with AAV-THAP11-29Q or AAV-THAP11-47Q and treated with control or PLX5622 diet. (**F**) Quantification of Western blotting results in **E** (*n* = 6, 1-way ANOVA with Tukey’s post tests). *P < 0.05, **P < 0.01, ***P < 0.001, ****P < 0.0001. Data are presented as mean values ± SEM.

TREM2 is a pivotal regulator of microglial state, but its effects on neurodegeneration appear to be dependent on the disease contexts (37–40). To determine whether TREM2 affects mutant THAP11 neuropathology, we performed stereotaxic injection of AAV-THAP11-29Q or AAV-THAP11-47Q into the cerebellum of 3-month-old WT or *Trem2* KO mice and sacrificed the mice 1 month later. The expression of NeuN, PSD95, PKCγ, and calbindin was unchanged in WT or *Trem2* KO mice injected with AAV-THAP11-29Q. In contrast, the reduction of NeuN, PSD95, PKCγ, and calbindin caused by THAP11-47Q was significantly attenuated in the cerebellum of *Trem2* KO mice compared with WT mice (Figure 8, A and B). We also performed immunofluorescence staining and found that the Purkinje cells in *Trem2* KO mice had a less degenerative morphology than in WT mice in the presence of THAP11-47Q (Figure 8C). Through Nissl staining, we confirmed that THAP11-47Q caused significantly less Purkinje cell loss in *Trem2* KO mice than in WT mice (Supplemental Figure 12, A and B). To further validate the role of microglia in SCA51 pathogenesis, we depleted microglia in the brains of 3-month-old WT mice injected with AAV-THAP11-29Q or AAV-THAP11-47Q using a standard AIN-76A rodent diet formulated with PLX5622 (Figure 8D), a selective CSF1R inhibitor (41, 42). The depletion of microglia in the cerebellum of the treated mice was confirmed 1 month later (Supplemental Figure 12C). Importantly, the reduction in NeuN, PSD95, PKCγ, and calbindin levels induced by THAP11-47Q was significantly alleviated in the cerebellum of mice treated with PLX5622 compared to those on the control diet (Figure 8, E and F). Immunofluorescence and Nissl staining revealed improved Purkinje cell survival in the cerebellum of mice injected with AAV-THAP11-47Q and treated with PLX5622 (Supplemental Figure 12, D–F). Collectively, these findings indicate that TREM2 deficiency or microglial depletion attenuates neurodegeneration caused by mutant THAP11 with polyQ expansion.

## Discussion

SCAs represent a group of heterogeneous inherited neurodegenerative disorders characterized by the typical cerebellum ataxia and other variable nonmotor symptoms. *THAP11* has been suspected as a causative gene for SCAs (43, 44), but definitive evidence linking *THAP11* with SCA51 was only made available recently (8), which establishes SCA51 as the tenth polyQ disease ever discovered. Given that SCA51 is an autosomal dominant disease and THAP11 is an important transcription factor, SCA51 offers a unique opportunity to investigate how polyQ expansion induces transcriptional dysregulation and brain region–specific neurodegeneration.

Our findings that expressing mutant THAP11 with polyQ expansion, but not reducing endogenous THAP11, leads to cerebellar neuronal damage strongly indicate that SCA51 is caused primarily by gain-of-function mechanisms. THAP11 is essential for the pluripotency of mouse embryonic stem cells, and deletion of *Thap11* leads to embryonic lethality in mice (12). However, the expression of THAP11 gradually declined in the brain as the animals matured into adulthood. These results suggest that the function of WT THAP11 may be different at various developmental stages. It would be interesting to investigate if mutant THAP11 with polyQ expansion causes early developmental deficits in mice.

As the causative gene for SCA51 was only recently identified, we packaged the *THAP11* gene carrying 47 CAG repeats from 1 SCA51 patient into AAVs and ectopically expressed mutant THAP11 in the mouse brain. One recent study established transgenic mouse models overexpressing WT THAP11 in the cerebellar Purkinje cells (45). The transgenic mice showed Purkinje cell loss and ataxia as early as 10 weeks of age (45). In contrast, our results showed that although THAP11-29Q and THAP11-47Q were expressed at comparable levels, only THAP11-47Q elicited neuronal damage in the cerebellum and the corresponding motor deficits. This discrepancy could be due to variations in the dose of gene overexpression. According to the Western blotting results of our study, the expression level of exogenous THAP11 is similar to endogenous THAP11 in the cerebellum, whereas the transgenic mouse model exhibits an about 10-fold increase in *Thap11* transcripts based on reverse transcriptase PCR analysis. An alternative explanation is that we expressed exogenous THAP11 specifically in the adult brain, but THAP11 is overexpressed throughout development in the transgenic mice so that the severe phenotypes of the transgenic mice could be derived from certain developmental effects.

Through transcriptomic analysis, we compared the overall gene expression in the cerebellum of WT mice that were either uninjected or injected with THAP11-29Q or THAP11-47Q. The transcriptome of the THAP11-29Q–expressing tissues closely resembled the uninjected tissues, which again demonstrated that overexpression of WT THAP11 is not deleterious in the adult cerebellum. Interestingly, when comparing the THAP11-29Q– and THAP11-47Q–expressing samples, we did not find global transcriptional disruptions, suggesting that the polyQ expansion only affects THAP11 in regulating a specific set of genes. Among the identified DEGs, we found a remarkable enrichment of marker genes related to microglial activation, including *Trem2* and its downstream targets (25, 26). TREM2 is specifically expressed in microglia, and its expression is increased in various neurodegenerative diseases (46–48). TREM2 modulates microglial functions in response to neuronal damage, but its exact role appears to be context dependent (16, 49). Specifically, multiple studies reported inconsistent roles of TREM2 signaling in neurodegeneration of β-amyloid toxicity (37, 50–54) or tauopathy (39, 40, 55). It is likely that there is a heterogenous population of TREM2-expressing microglia that play distinct functions depending on disease stages and misfolded proteins (56).

We established a KI mouse model of SCA51, utilizing the genomic DNA directly obtained from a patient diagnosed with SCA51. A CAG repeat expansion of over 100 is typically necessary for KI mice to exhibit obvious neurological phenotypes (24, 29, 30, 57). Therefore, to leverage single-cell sequencing for uncovering the transcriptional signatures of Purkinje and granule cells in SCA51 pathogenesis, aged THAP11-47Q KI mice or another KI mouse model expressing THAP11 with longer CAG repeats are needed. Nonetheless, the THAP11-47Q KI mouse model is ideal for studying the pathogenic mechanisms that precede neuronal loss. We discovered that the expression of TREM2 and its downstream targets was upregulated in the THAP11-47Q KI mice, suggesting that the endogenous expression of mutant THAP11 elicits microglial activation. The observed microglial activation could be attributed to either inflammatory responses from neuronal insult or an inherent reaction independent of any external stimulus. Our results in HMC3 cells suggest that mutant THAP11 binds to the *TREM2* promoter, thereby triggering its activation. However, to validate this in THAP11-47Q KI mice, it is necessary to develop an antibody capable of immunoprecipitating mouse glial THAP11 to perform a ChIP assay and investigate the in vivo regulation of *TREM2* by THAP11. Alternatively, generating a conditional KI mouse model that specifically expresses mutant THAP11 in microglia would offer definitive validation of this finding. In *Trem2* KO mice and WT mice with microglial depletion, neuronal damage induced by mutant THAP11 was attenuated, suggesting that TREM2-mediated microglial responses contribute to mutant THAP11 toxicity in the brain. However, we cannot rule out the possibility that mutant THAP11 is also intrinsically neurotoxic within neurons.

In summary, our study provides insight into the pathogenic mechanisms of SCA51 using animal models. The absence of neuronal loss from THAP11 knockdown suggests the potential for developing THAP11-lowering strategies as a treatment approach for SCA51. The obvious protein aggregation and neuropathological phenotypes caused by THAP11-47Q indicate that SCA51 could be an ideal model for understanding brain region–specific neurodegeneration. Our findings also indicate that TREM2-mediated microglial activation is involved in selected polyQ diseases, but the activation process and consequence may vary depending on the disease context, which warrants future studies.

## Methods

### Sex as a biological variable.

Sex was not considered as a biological variable.

### Animals.

WT mice (C57BL/6J strain) were purchased from Guangzhou Yancheng Biotechnology. *Trem2* KO mice (C001207) were purchased from Cyagen. The THAP11-47Q KI mouse model in the C57BL/6J background was generated by GemPharmatech. Cre-dependent Cas9 transgenic mice (Jackson Laboratory; strain 024857) were crossed with E2a-Cre transgenic mice (Jackson Laboratory; strain 003724) to generate germline-transmissible mice that ubiquitously expressed Cas9 in all tissues. The mice were maintained in a 12-hour light/dark cycle at the Division of Animal Resources of Jinan University.

### Antibodies and plasmids.

The following primary antibodies were used in this study: THAP11 (Proteintech; 23030-1), calbindin (Proteintech; 14479-1), PKCγ (Proteintech; 14364-1), NeuN (Abcam; ab177487 and ab104224), IBA1 (Wako, 019-19741; Abcam, ab5076), GFAP (Sigma; G3893), Vinculin (Sigma; MAB3574), GFP (Abcam; 13970), 1C2 (Millipore; MAB1574), HA (Proteintech; 51064-2), zsGreen (Sangon Biotech; D199984-0100), Cas9 (Abcam; 191468), PSD95 (Abcam; 238135), TREM2 (Santa Cruz Biotechnology, sc-373828; Cell Signaling Technology, 91068; Aves Labs, TREM2), C1qA (HUABIO; HA721439), C1qC (Sangon Biotech; D123951), Clec7A (Sangon Biotech; D220382), ATXN3 (Millipore; MAB5360), and HTT (Sigma; MAB5374).

Thap11-siRNAs were purchased from GenePharma, and the sequences are listed as follows: Thap11-siRNA1 sense, 5′-GCGCUGCACUUCUACACGUTT-3′, antisense, 5′-ACGUGUAGAAGUGCAGCGCTT-3′; Thap11-siRNA2 sense, 5′-GGCUGUCAUCCGUAAGAAGTT-3′, antisense, 5′-CUUCUUACGCAUGACAGCCTT-3′; and Scramble ctrl-siRNA sense, 5′-UUCUCCGAACGUGUCACGUTT-3′, antisense, 5′-ACGUGACACGUUCGGAGAATT-3′.

To construct the Thap11-gRNA plasmids, 2 gRNA sequences were used: gRNA1, 5′-ACGCTGAGTTGCGGCGCCTC-3′, and gRNA2, 5′-GCTGCAGAGACGGTGGCCGG-3′. The 2 gRNA sequences were inserted into the ssAAV.U6.sgRNA.EFS.zsGreen plasmid (purchased from PackGene Biotech). To construct the THAP11-29Q and THAP11-47Q plasmids, the genomic DNAs from HEK293 cells and one SCA51 patient were used as templates for PCR amplification. The sequences of the primers used are listed as follows: sense, 5′-CACCGGTGCCACCATGGGATACCCATACGATGTTCCAGATTACGCTATGCCTGGCTTT-3′, and antisense, 5′-AGAGAAGTTTGTTGCGCCGGATCCCATTCCGTGCTTCTTGCGGA-3′. The amplified DNAs were inserted into the ssAAV.CMV plasmid (purchased from PackGene Biotech). The ATXN3 plasmids were gifts from Olaf Riess at the University of Tübingen. The HTT exon 1 plasmids were described in our previous study (58).

### Cell culture and MTT assay.

HEK293, HMC3, and N2a cells were purchased from ATCC and cultured in DMEM (Gibco; C11995500CP) containing 10% fetal bovine serum (Biological Industries; 04-001-1B) and 100 U/mL penicillin and streptomycin (Gibco; 15140163). The culture medium was replaced every 2 days. For transient transfection of plasmids or siRNAs, the cells were grown to 70%–80% confluency, and lipofectamine 3000 (L3000001; Invitrogen) was used according to the manufacturer’s instructions. The cells were harvested 48 hours after transfection. To perform the MTT assay, the cells were collected, and the MTT Cell Proliferation and Cytotoxicity Assay Kit (Solarbio; M1020) was used according to the manufacturer’s protocol. A BioTek EPOCH microplate spectrophotometer was used to measure the optical density.

### Virus packaging and stereotaxic surgery.

All the AAV plasmids were packaged into AAV serotype 9 at a concentration of 1 × 10^13^ GC/mL by PackGene Biotech. For stereotaxic surgery, the mice were anesthetized with continuous inhalation of 1.5% isoflurane and fixed on a mouse stereotaxic apparatus (RWD; 69100). The hair surrounding the surgical site was removed, and the skin was disinfected with 75% alcohol. The injection site was determined based on the coordinates from bregma: striatum, anterior-posterior = 0.55 mm, medial-lateral = ± 2 mm, dorsal-ventral = 3.4 mm; cerebellum, anterior-posterior = –6.3 mm, medial-lateral = ± 1.7 mm, dorsal-ventral = 1.5 mm. A microdrill was used to make small holes in the skull, and the AAVs were injected into the brain using a 33G microsyringe (Hamilton; NanoFil-10ul-1) at a speed of 200 nL/min. After the injection was completed, the needle was kept in the brain for 10 minutes and then slowly withdrawn. The wound was sutured, and the mouse was placed on a warm blanket until awakened.

### Mouse behavioral tests and drug administration.

For the rotarod test, the mice were trained on the rotarod at 5 rpm for 5 minutes, 3 times daily for 3 consecutive days. In the actual test, the speed of the rotarod was set to slowly accelerate from 5 to 40 rpm over a period of 5 minutes. The time of the mice staying on the rotarod was recorded. The result was the average of 3 trials. For the balance beam test, the mice were placed on a 100 cm long, 6 mm wide beam hanging 50 cm above the floor. The mice were trained to move from one end of the beam to the other 3 times daily for 3 consecutive days. In the actual test, the time that the mice moved 80 cm on the beam was recorded. The result was the average of 3 trials. For the grip strength test, the mice were allowed to grip the metal grids of a grip meter with all their limbs, and they were gently pulled backwards by the tail until they could no longer hold the grids. For the gait analysis, the CatWalk XT system (Noldus) was used for the acquisition of the footprints and the corresponding quantitative assessment. Each group contained age- and sex-matched mice. The tests were performed by personnel who were blind to the experimental groups.

To pharmacologically deplete microglia in the brain, the mice were fed a PLX5622-formulated AIN-76A diet (1.2 g PLX5622 per kilogram of diet; MedChemExpress) ad libitum. Control mice were fed a standard AIN-76A diet.

### Western blotting and filter trap assays.

The brain tissues were dissected and ground in RIPA lysis buffer (Beyotime; P0013C) by a Luka grinding instrument (LUKYM-II). The protein concentration was determined using a BCA Protein Assay Kit (GBCBIO Technologies; G3522). Equal amounts of proteins were used for SDS-PAGE (GenScript; M00652). The blots were blocked with 5% skim milk for 2 hours, and the primary antibody was added and incubated at 4°C overnight. On the following day, the blots were washed 3 times with 1× PBS, incubated with the secondary antibody for 2 hours, and then washed 3 times with 1× PBS. The blots were developed with ECL solution (Millipore; WBKLS0500), and the images were acquired digitally using a Clinx ChemiScope 6300.

For filter trap assays, the brain lysate was passed through cellulose acetate membranes with 0.22 μm pore size (Solarbio; YA0672) using a 96-well dot blot apparatus. The proteins captured by the cellulose acetate membranes were detected using the same methods as those employed for Western blotting.

### Immunohistochemistry and immunofluorescence staining.

The mice were anesthetized with isoflurane and perfused with 0.9% NaCl solution followed by cold 4% paraformaldehyde (PFA). The brains were dissected and fixed in 4% PFA at 4°C overnight. Then the fixed brains were dehydrated in 15%–30% sucrose solution for 2 days. The brains were embedded in OCT (Sakura; 4583) and sectioned in a cryostat (CryoStar NX50, Thermo Scientific).

For immunohistochemistry, the brain slices were rinsed 3 times in 1× PBS and incubated in 0.3% H_2_O_2_ for 10 minutes. The slices were blocked in the blocking buffer (3% BSA, 2% goat serum, 0.1% Triton X-100, 1× PBS) for 1 hour and incubated with primary antibodies in the blocking buffer at 4°C overnight. On the following day, the remaining steps were performed according to the instructions of the Mouse and Rabbit Specific HRP/DAB (ABC) Detection IHC kit (Abcam; ab64264).

For immunofluorescence staining, the brain slices were rinsed in 1× PBS, blocked in the blocking buffer, and incubated with primary antibodies in the blocking buffer at 4°C overnight. On the following day, the slices were rinsed with 1× PBS and incubated with fluorescent secondary antibodies at room temperature for 1 hour. The fluorescent images were acquired using a Zeiss AX10 Axio microscope or Olympus FV3000 confocal laser scanning microscope. ImageJ (NIH) software (version 1.54d) was used for the quantification of staining intensity. The image type was converted to 8-bit grayscale. The “threshold” function was used to subtract background, and the “measure” function was used to quantify the staining intensity. The Sholl analysis plug-in (https://imagej.net/plugins/sholl-analysis) was used to analyze microglial morphology.

Nissl staining was performed using a cresyl violet kit (Solarbio; G1430) following the manufacturer’s instructions. Briefly, the brain slices were immersed in cresyl violet solution for 1 hour, washed with water, and differentiated until the background turned white.

### RNA-Seq.

The RNAs from mouse cerebellum tissues were extracted using an RNeasy Lipid Tissue Mini Kit (Qiagen; 74804). The RNAs were sent to Novogene for library preparation and sequencing. The raw data were quantified using Salmon software (version 1.9.0) with mapping-based mode. The DEGs were analyzed using the edgeR package (version 3.1.6) with adjusted *P* value < 0.05 and |log_2_Fold Change| > 0.75. The correlation map was plotted using the corrplot (version 0.92) package. To explore the related GO and Kyoto Encyclopedia of Genes and Genomes pathways, the clusterProfiler (version 4.0.2) package was used for the upregulated or downregulated genes with *P* value < 0.05 and |log_2_Fold Change| > 0.75. The ComplexHeatmap package (version 2.16.0) was used to draw the heatmap plot. The GSVA package (version 1.5.0) was used to estimate the variations of gene set enrichment. All analyses were performed using R (version 4.3.0) and Rstudio (Ver2023.09.1+494) software.

### Quantitative real-time PCR.

The extracted RNAs were reverse transcribed into cDNAs using a PrimeScript RT Reagent Kit with gDNA Eraser (Takara; RR047A). SYBR Green PCR Master Mix (Qiagen; 208352) and the CFX Connect Real-Time PCR System (Bio-Rad) were used for quantitative real-time PCR. The primers used are listed as follows: *Trem2*, forward 5′-TTTCTTGCAGCCAGCATCCTC-3′, reverse 5′-TCTCACGTACCTCCGGGTCCA-3′; *Clec7A*, forward 5′-ATTGAAAGCCAAACATCGTCT-3′, reverse 5′-CTTCACTCTGATTGCGGGAA-3′; *Lyz2*, forward 5′-GTGTAATGATGGCAAAACCCC-3′, reverse 5′-CTCTTTGCACATTGTATGGCT-3′; *Lpl*, forward 5′-TCTAACTGCCACTTCAACCAC-3′, reverse 5′-CTCATACATTCCCGTTACCG-3′; *Ccl6*, forward 5′-CTGCTTCTCTTATGCCACA-3′, reverse 5′-TTAGGCACCTCTGAACTCT-3′; *C1qc*, forward 5′-GCACCTGAACCTCAACCTTGCC-3′, reverse 5′-TAGCCACACCTCATCGCCCCTC-3′.

### ChIP.

HMC3 cells were transfected with THAP11 plasmids and collected 2 days after transfection. The cells were fixed with 1% formaldehyde for 10 minutes at 37°C and transferred to SDS lysis buffer. Genomic DNA was sheared by 10 seconds × 10 times sonication. ChIP was performed according to the ChIP assay kit manual (ABclonal; RK20258). THAP11 antibody was used to pull down THAP11 and its binding DNA. Primers used for PCR amplification of the *TREM2* promoter are listed as follows: forward 5′-CAGCCTCTTCTGCCACTCC-3′ and reverse 5′-CCCATGGTGATCCAAGCACAG-3′.

### Luciferase assay.

Human *TREM2* promoter (–1,500–0 bp) was isolated from genomic DNA by PCR with forward primer 5′-CGGGGTACCGGTGGTTGTTATAGAGATGCATGAGT-3′ and reverse primer 5′-CCCAAGCTTGCCACCCTTCCCCAGCCAAG-3′. The isolated promoter sequence was inserted into the pGL4.14 plasmid (Promega) using HindIII and KpnI restriction sites. The luciferase reporter and THAP11 plasmids were transfected into HMC3 cells. Two days after transfection, the cells were collected and the luciferase assay performed using the ONE-Glo Luciferase Assay System (Promega). Luciferase intensity was measured by a BioTek Synergy H4 microplate reader (Agilent BioTek).

### Statistics.

The data were analyzed using Prism 9 software (GraphPad). For comparisons between 2 groups, 2-tailed Student’s *t* tests were used. For 3 or more groups, 1-way ANOVA with Tukey’s multiple-comparison tests were used. All experiments were repeated at least 3 times, and the quantification was presented as mean ± SEM. A *P* value of less than 0.05 was considered significant.

### Study approval.

Mouse breeding and procedures were submitted to the IACUC of Jinan University under application number 12806 and approved under approval number IACUC-20210220-06.

### Data availability.

The raw RNA-Seq data have been uploaded to the BioProject database with accession PRJNA1040027. Values for all data points in graphs are reported in the Supporting Data Values file. The data generated in this study are available upon request from the corresponding authors.

## Author contributions

Su Yang and HJ conceived the project. Su Yang designed research. ER, JL, ZC, QS, LC, JH, XD, YQ, TX, Sitong Yang, and MP performed research. XG and PY provided key research samples and experimental techniques. Su Yang and ER analyzed results and wrote the manuscript. XJL, SL, and HJ edited the manuscript. ER, JL, and ZC made significant contributions to this study, justifying their co–first authorship status. The order of authorship reflects their respective contributions.

## Supplementary Material

Supplemental data

Unedited blot and gel images

Supplemental video 1

Supporting data values

## Version 1 06/03/2025

In-Press Preview

## Version 2 07/15/2025

Electronic publication
