# Supplementary material for: Mutant THAP11 causes cerebellar neurodegeneration and triggers TREM2-mediated microglial activation in mice
Source: J Clin Invest. 2025 Jun 3;135(14):e178349. doi: 10.1172/JCI178349 (PMC12259261; doi:10.1172/JCI178349)
Supplement: Supplemental data [file jci-135-178349-s180.pdf]

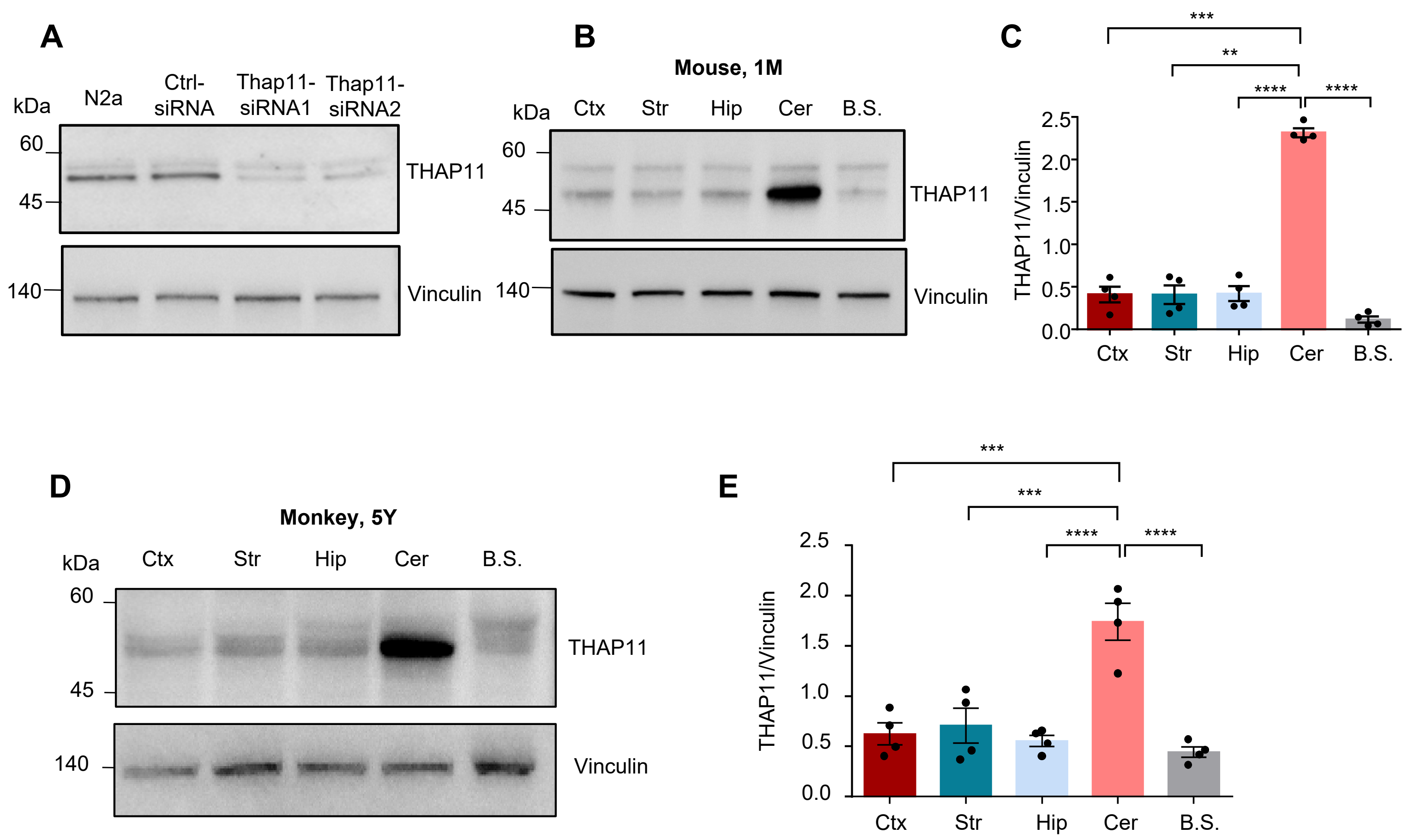

Supplemental Figure 1. Further characterization of THAP11 expression in the brain (related to Figure 1). (A) Western blotting of THAP11 in the N2a transfected with control Thap11 siRNA. **(B)** Western blotting of THAP11 in different brain regions of 1-month-old mice (Ctx, cortex; Str, striatum; Hip, hippocampus; Cer, cerebellum; B.S., brain stem). **(C)** Quantitative results of Supplemental Figure 1B ($n = 4$, one-way ANOVA with Tukey's post-tests). **(D)** Western blotting of THAP11 in different brain regions of 5-year-old monkeys. **(E)** Quantitative results of Supplemental Figure 1D ($n = 4$, one-way ANOVA with Tukey's post-tests). $^{**}P < 0.01$, $^{***}P < 0.001$, $^{****}P < 0.0001$. Data are presented as mean values ± SEM.

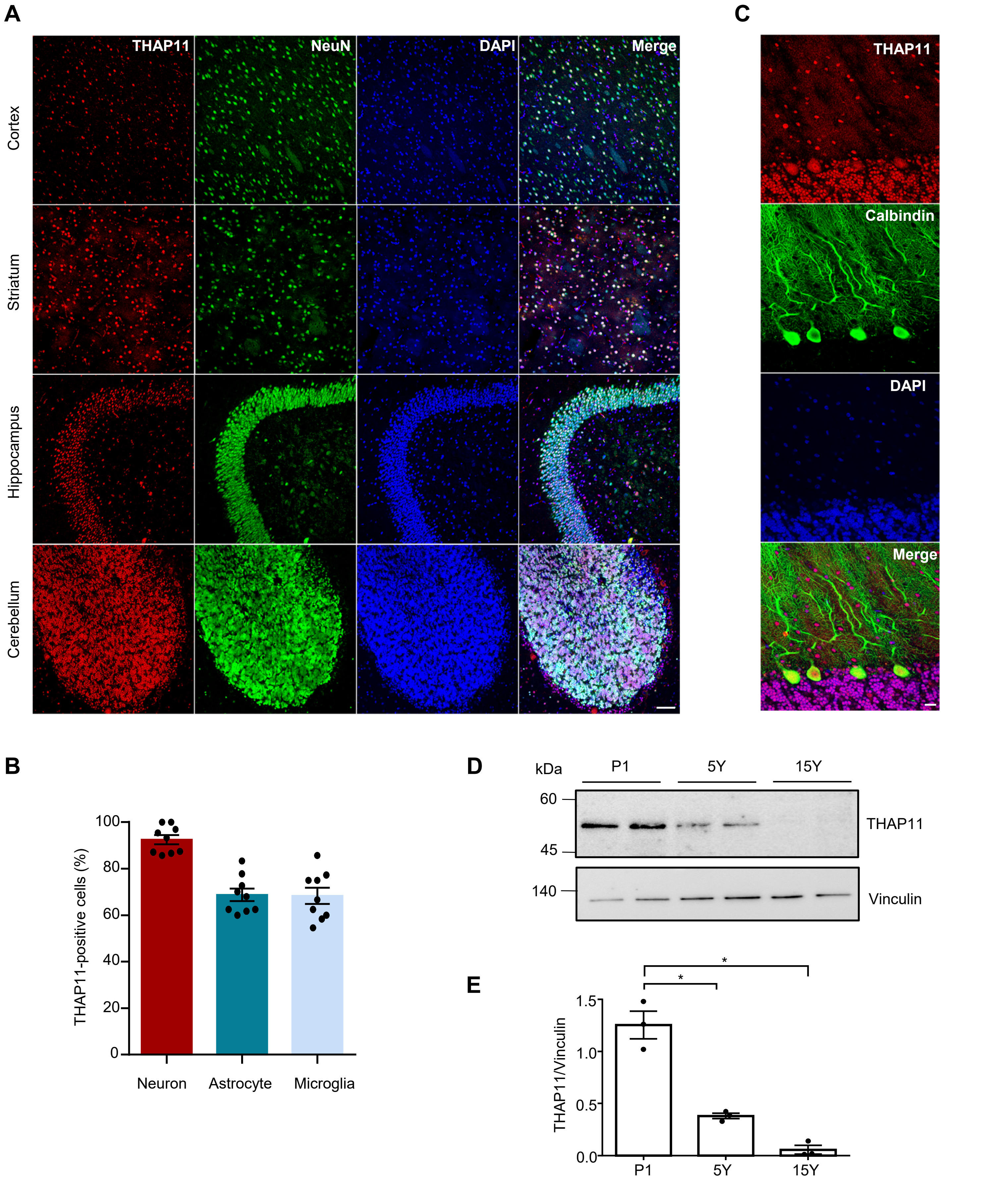

Supplemental Figure 2. THAP11 staining in the brain (related to Figure 1). (A) Immunofluorescent staining of THAP11 and NeuN in the monkey brain (scale bar: 100 μm). **(B)** Percentage of neurons, astrocytes and microglia that are THAP11-positive (*n* = 9). **(C)** Immunofluorescent staining of THAP11 and calbindin in the cerebellum (scale bar: 20 μm). **(D)** Western blotting of THAP11 in the monkey cerebellum at different ages (P1, postnatal day 1; 5Y, 5-year-old; 15Y, 15-year-old). **(E)** Quantitative results of Supplemental Figure 2D (*n* = 3, one-way ANOVA with Tukey's post-tests). $^{*}P < 0.05$. Data are presented as mean values ± SEM.

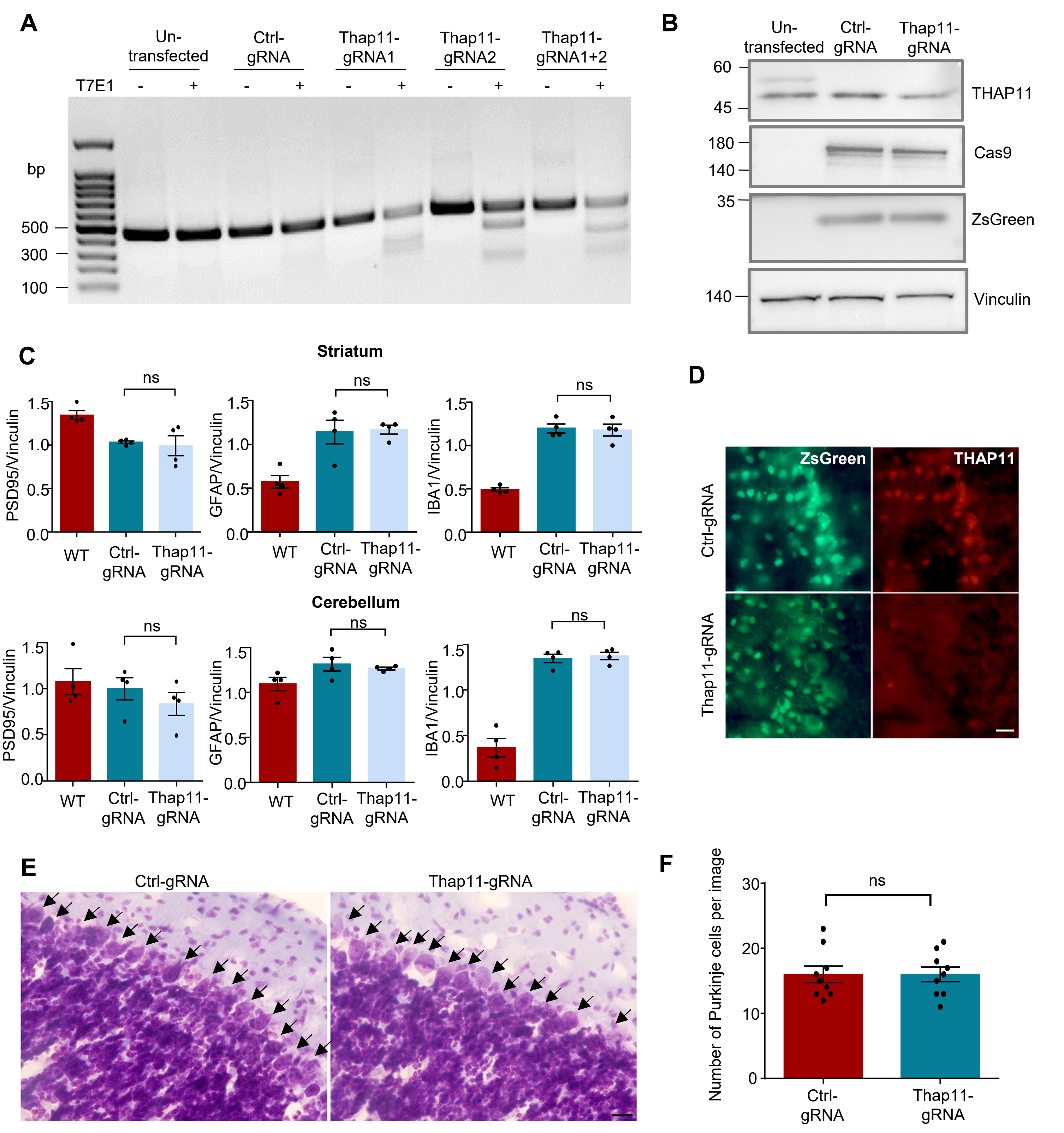

Supplemental Figure 3. Verification of THAP11 knockdown by CRISPR/Cas9 genome editing (related to Figure 2). (A) T7E1 assay was performed to test the targeting efficiency of Thap11 gRNAs in N2a cells. **(B)** Western blotting analysis of THAP11 in the N2a cells that were transfected with Cas9 and gRNA plasmids. Vinculin served as a loading control. **(C)** Quantification of PSD95, GFAP, and IBA1 staining intensity in Figure 2B (*n* = 4, one-way ANOVA with Tukey's post-tests). **(D)** Immunofluorescent staining of THAP11 in the brain of germline Cas9 mice injected with AAV-Ctrl-gRNA or AAV-Thap11-gRNA. ZsGreen fluorescence reflected the AAV-infected cells (scale bar: 20 μm). (**E)** Nissl staining images of cerebellum slices form germline Cas9 mice injected with AAV-Ctrl-gRNA or AAV-Thap11-gRNA (scale bar: 20 μm). **(F)** Quantification of Purkinje cell number in Supplemental Figure 3E (*n* = 9 from three mice, two-tailed student t-test). Ns, non-significant. Data are presented as mean values ± SEM.

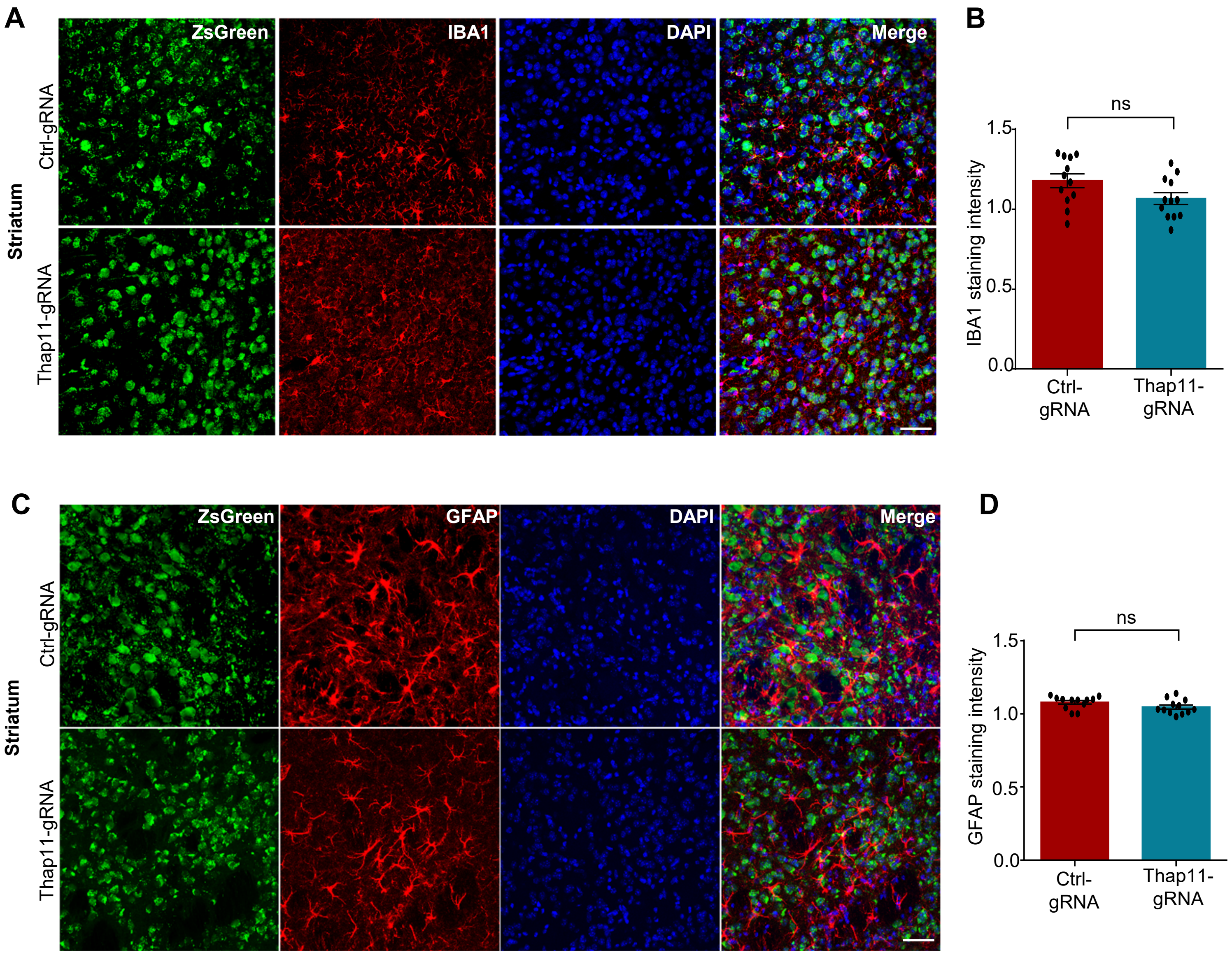

Supplemental Figure 4. THAP11 knockdown does not cause neuronal damage in the striatum (related to Figure 2). (A) Immunofluorescent staining of IBA1 in the striatum of germline Cas9 mice injected with AAV-Ctrl-gRNA or AAV-Thap11-gRNA. ZsGreen fluorescence reflected the AAV infected cells (scale bar: 50 μm). **(B)** Quantification of IBA1 staining intensity in Supplemental Figure 4A (*n* = 12 from three mice, two-tailed student t-test). **(C)** Immunofluorescent staining of GFAP in the striatum of germline Cas9 mice injected with AAV-Ctrl-gRNA or AAV-Thap11-gRNA. ZsGreen fluorescence reflected the AAV infected cells (scale bar: 50 μm). **(D)** Quantification of GFAP staining intensity in Supplemental Figure 4C (*n* = 12 from three mice, two-tailed student t-test). Ns, non-significant. Data are presented as mean values ± SEM.

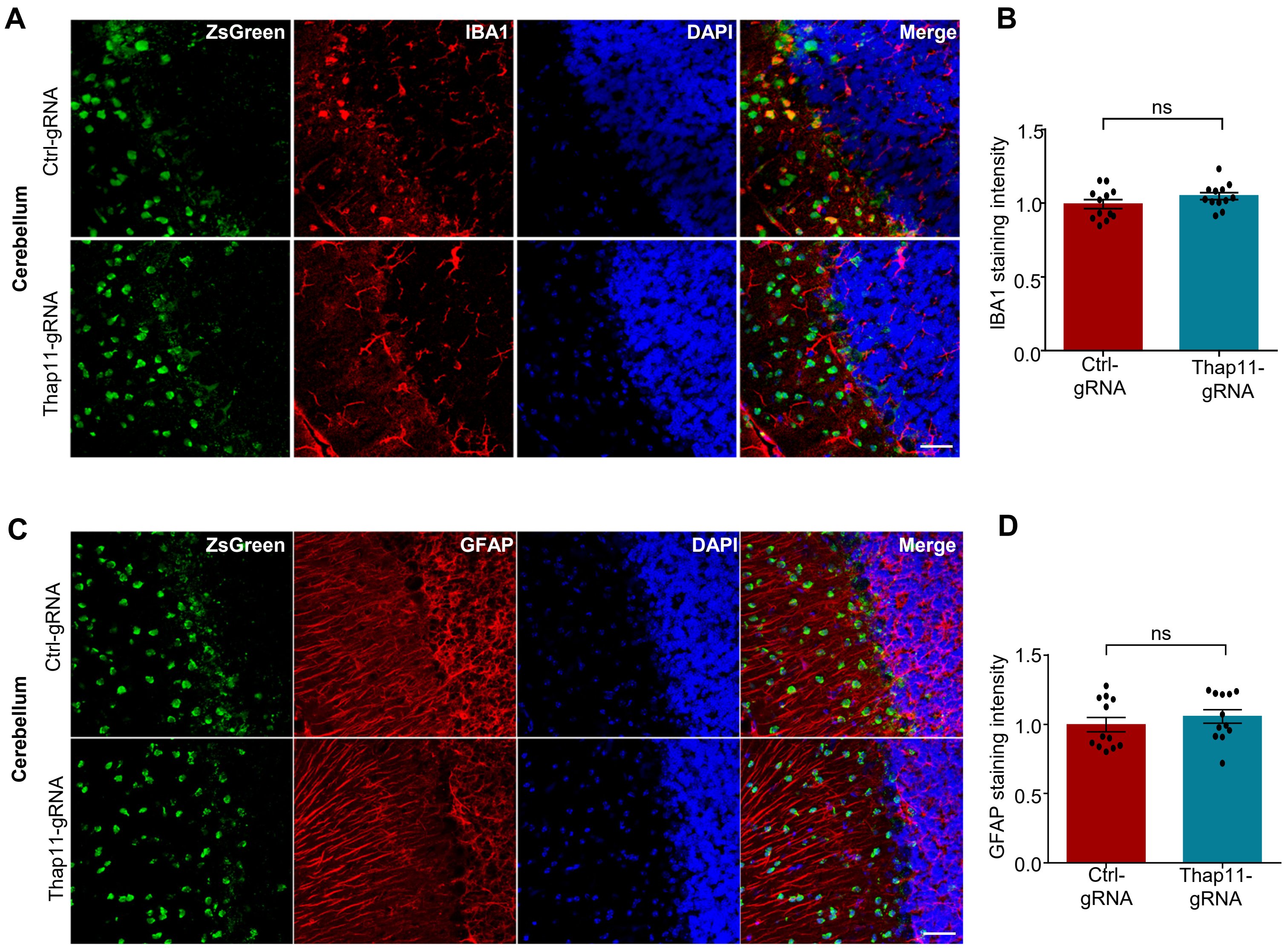

Supplemental Figure 5. THAP11 knockdown does not cause neuronal damage in the cerebellum (related to Figure 2). (A) Immunofluorescent staining of IBA1 in the cerebellum of germline Cas9 mice injected with AAV-Ctrl-gRNA or AAV-Thap11-gRNA. ZsGreen fluorescence reflected the AAV infected cells (scale bar: 50 μm). **(B)** Quantification of IBA1 staining intensity in Supplemental Figure 5A (n = 12 from three mice, two-tailed student t-test). **(C)** Immunofluorescent staining of GFAP in the cerebellum of germline Cas9 mice injected with AAV-Ctrl-gRNA or AAV-Thap11-gRNA. ZsGreen fluorescence reflected the AAV infected cells (scale bar: 50 μm). **(D)** Quantification of GFAP staining intensity in Supplemental Figure 5C (n = 12 from three mice, two-tailed student t-test). Ns, non-significant. Data are presented as mean values ± SEM.

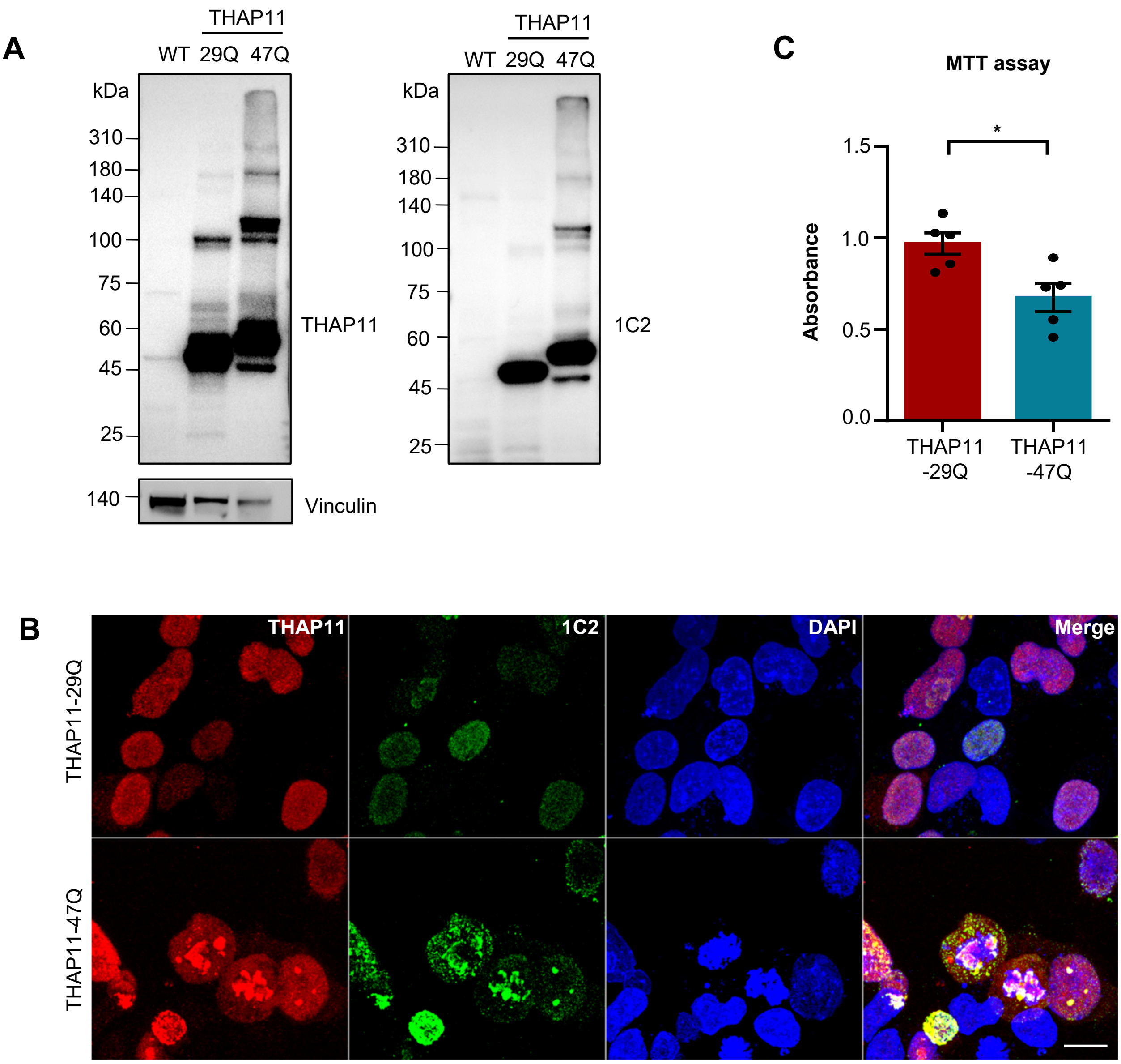

Supplemental Figure 6. Mutant THAP11 with polyQ expansion forms aggregates *in vitro* (related to Figure 3). (A) Western blotting analysis of THAP11 in HEK293 cells that were either untransfected, transfected with THAP11-29Q or THAP11-47Q plasmids. THAP11 and 1C2 antibodies were used to detect THAP11. **(B)** Immunofluorescent staining of THAP11 in the HEK293 cells transfected with THAP11-29Q or THAP11-47Q plasmids (scale bar: 10 μm). **(C)** MTT cell viability assay of HEK293 cells transfected with THAP11-29Q or THAP11-47Q plasmids (n = 5, two-tailed student t-test). *P < 0.05. Data are presented as mean values ± SEM.

Supplemental Figure 7

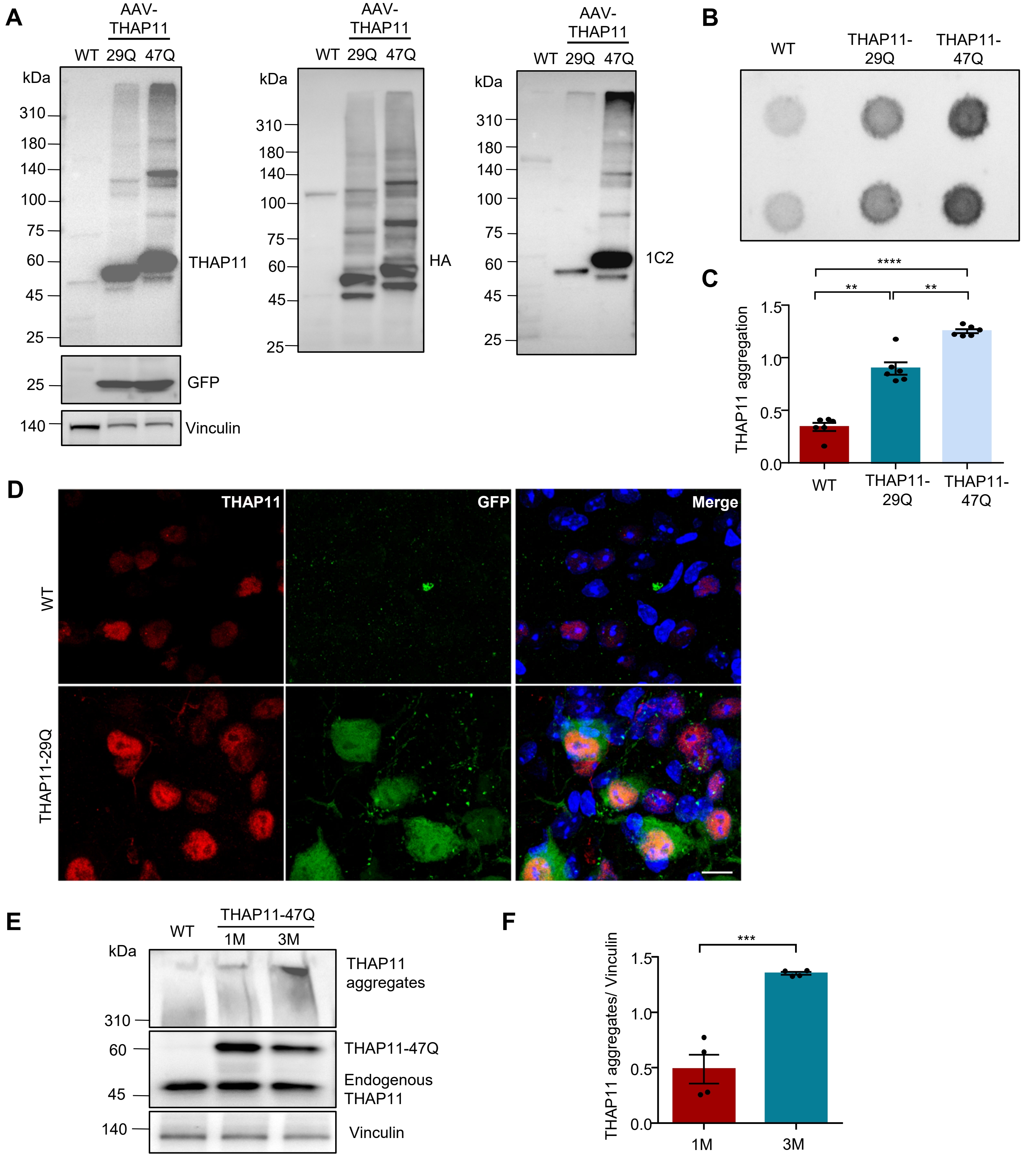

Supplemental Figure 7. Further validation of mutant THAP11 aggregation (related to Figure 3). (A) Western blotting analysis of THAP11 in HEK293 cells that were either untransfected, transfected with AAV-THAP11-29Q or AAV-THAP11-47Q plasmids. THAP11, HA and 1C2 antibodies were used to detect THAP11. **(B)** Filter trap assays were performed using the cerebellar lysates from WT mice or mice injected with AAV-THAP11-29Q or AAV-THAP11-47Q. **(C)** Quantitative results of Supplemental Figure 7B (*n* = 6, one-way ANOVA with Tukey's post-tests). **(D)** Immunofluorescent staining of THAP11 in the striatum of WT mice or mice injected with AAV-THAP11-29Q (scale bar: 20 μm). **(E)** Western blotting of THAP11 in 1 and 3-month-old mice that were injected with AAV-THAP11-47Q. **(F)** Quantitative results of Supplemental Figure 7E (*n* = 4, two-tailed student t-test). *$P < 0.05$, **$P < 0.01$, ***$P < 0.001$. Data are presented as mean values ± SEM.

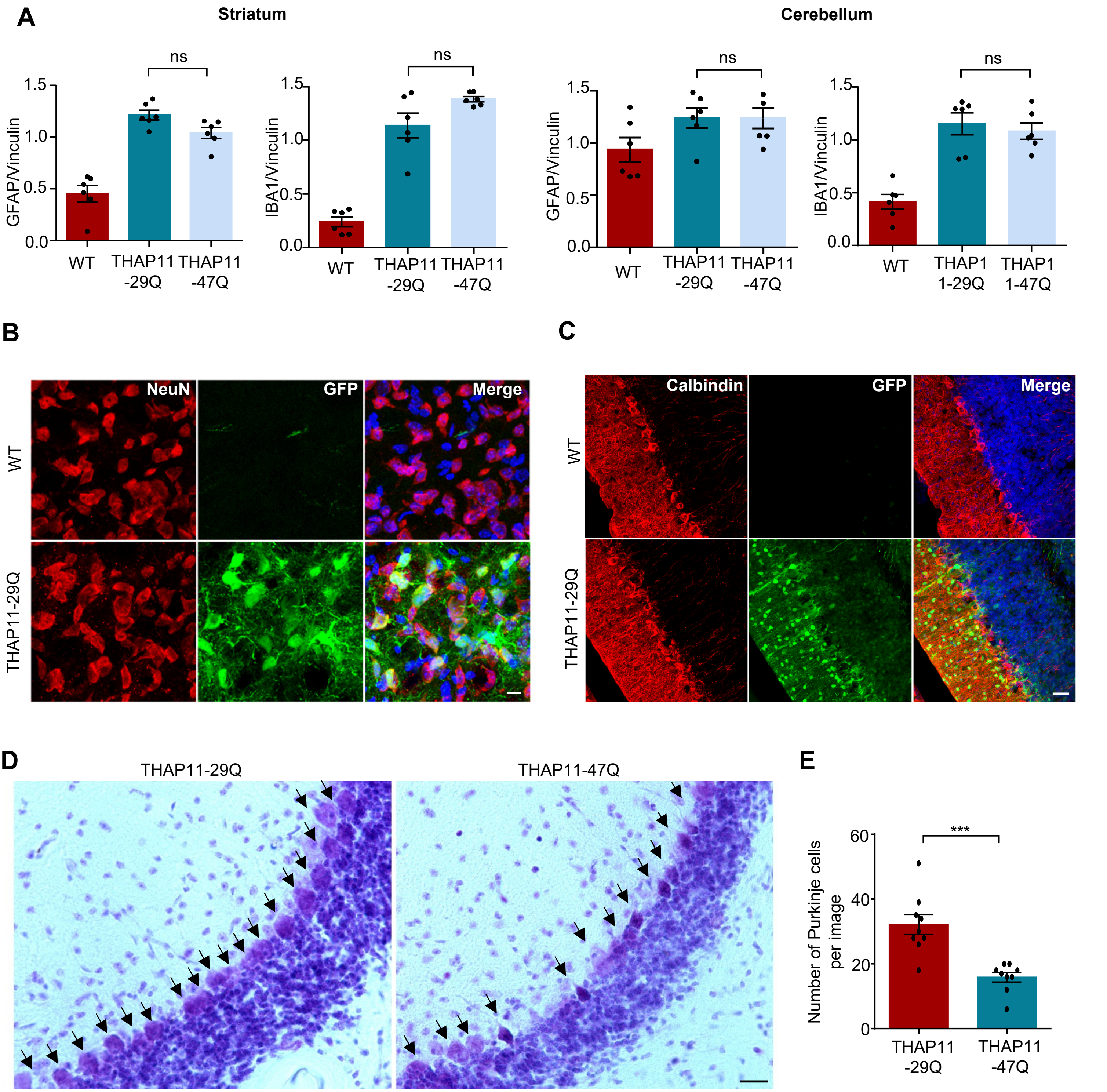

Supplemental Figure 8. THAP11-47Q causes Purkinje cell loss in the cerebellum (related to Figure 4). **(A)** Quantification of GFAP and IBA1 western blotting results in Figure 4A (*n* = 6, one-way ANOVA with Tukey's post-tests). **(B)** Immunofluorescent staining of NeuN in the striatum of WT mice and mice injected with AAV-THAP11-29Q (scale bar: 20 μm). **(C)** Immunofluorescent staining of calbindin in the cerebellum of WT mice and mice injected with AAV-THAP11-29Q (scale bar: 50 μm). **(D)** Nissl staining of cerebellum slices from mice injected with AAV-THAP11-29Q or AAV-THAP11-47Q (scale bar: 50 μm). **(E)** Quantification of Purkinje cell number in Supplemental Figure 8D (*n* = 9 from three mice, two-tailed student t-test). Ns, non-significant, ***$P < 0.001$. Data are presented as mean values ± SEM.

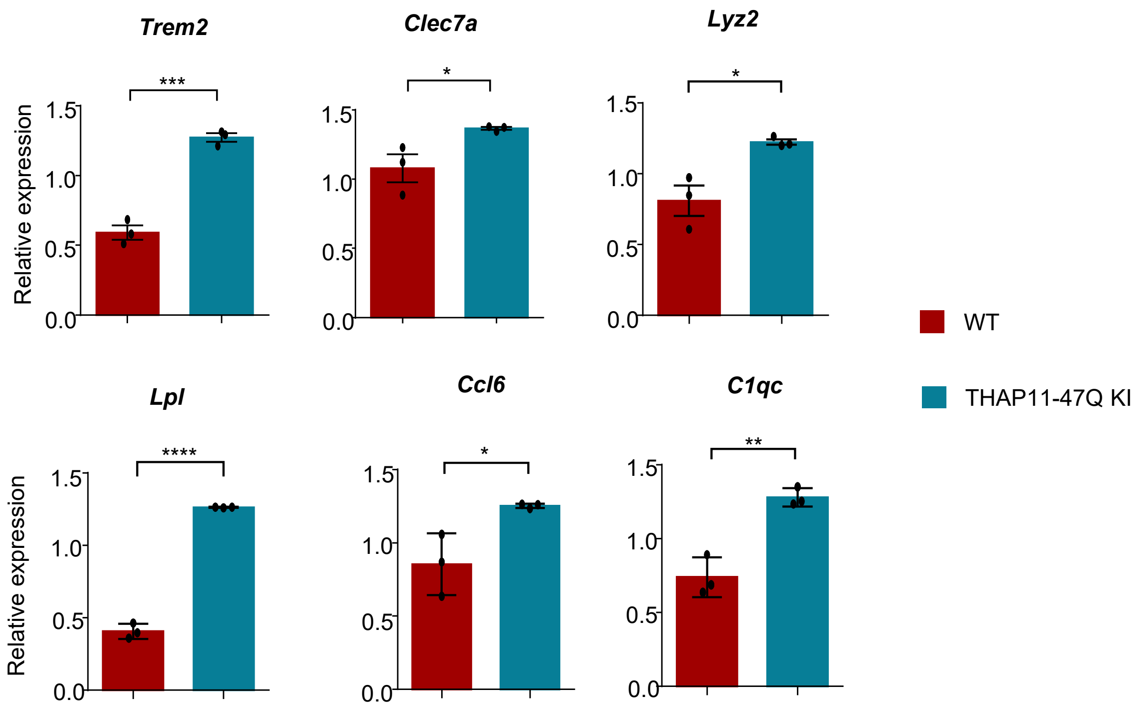

Supplemental Figure 9. Microglial activation in THAP11-47Q KI mice (related to Figure 7). Quantitative real-time PCR analysis of selected genes using the cerebellum of 7-month-old WT and THAP11-47Q KI mice (n = 3, two-tailed student t-test). *P < 0.05, **P < 0.01, ***P < 0.001, ****P < 0.0001. Data are presented as mean values ± SEM.

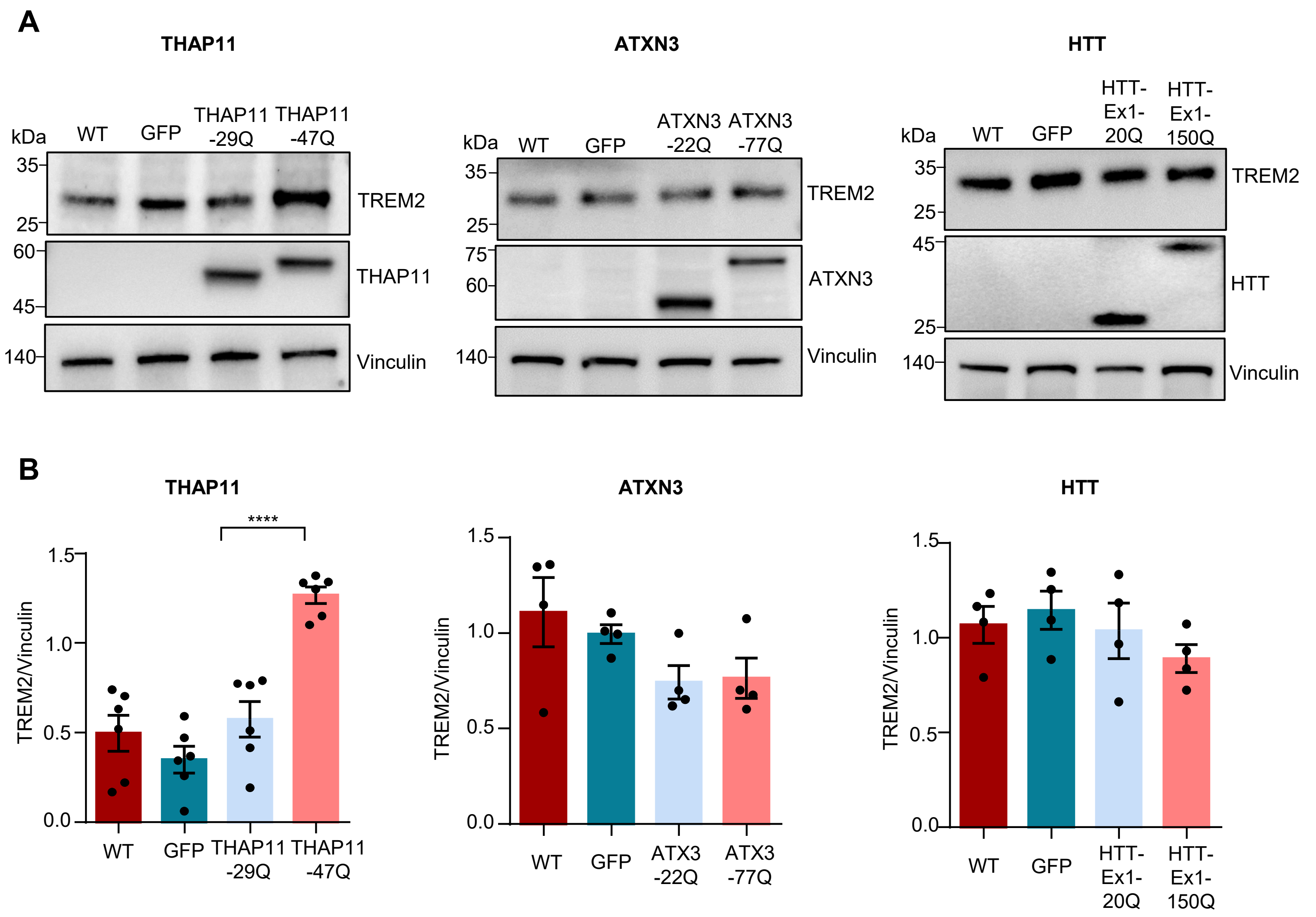

Supplemental Figure 10. TREM2 expression is induced specifically by mutant THAP11 (related to Figure 8). (A) Western blotting analysis of TREM2 in the HMC3 cells that were transfected with THAP11, Ataxin3 (ATXN3) or Huntingtin (HTT) plasmids. Vinculin served as a loading control. **(B)** Quantification of western blotting results in Supplemental Figure 10A (*n* = 4 - 6, one-way ANOVA with Tukey's post-tests). ****$P < 0.0001$. Data are presented as mean values ± SEM.

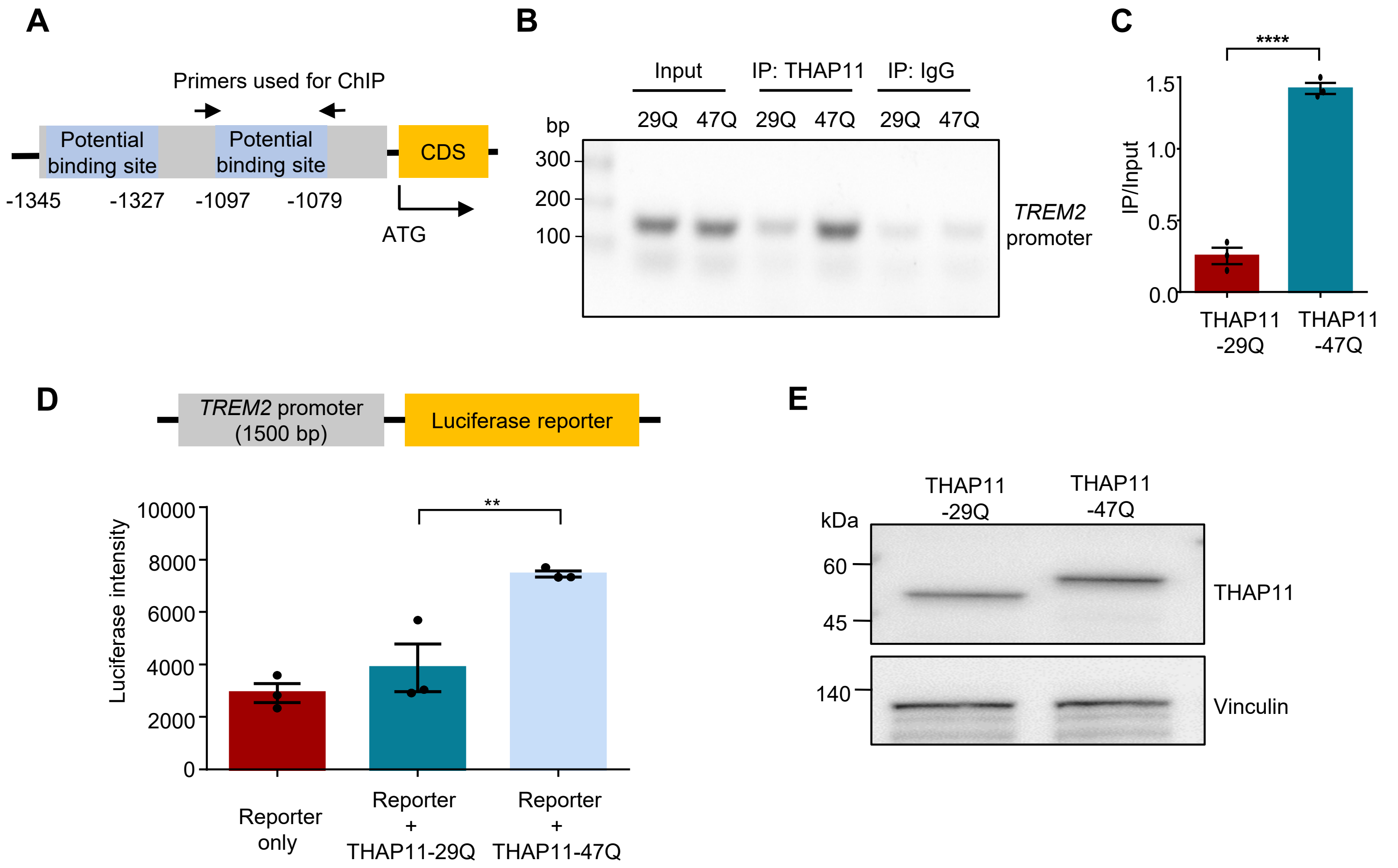

Supplemental Figure 11. THAP11-47Q upregulates *TREM2* transcription in HMC3 cells (related to Figure 8). (A) A schematic representation of potential THAP11 binding sites in *TREM2* promoter. CDS, coding sequence. **(B)** ChIP assay was performed using HMC3 cells transfected with THAP11-29Q or THAP11-47Q. PCR results showed the amount of *TREM2* promoter immunoprecipitated by THAP11. **(C)** Quantification of ChIP results in Supplemental Figure 11B (n = 3, two-tailed student t-test). **(D)** A schematic representation of the luciferase reporter controlled by *TREM2* promoter. Luciferase intensity of HMC3 cells transfected with the luciferase reporter and THAP11-29Q or THAP11-47Q was quantified (n = 4, one-way ANOVA with Tukey's post-tests). **(E)** Western blotting analysis of THAP11 expression in the HMC3 cells used for the luciferase assay. Vinculin served as a loading control. **$P < 0.01$, ****$P < 0.0001$. Data are presented as mean values ± SEM.

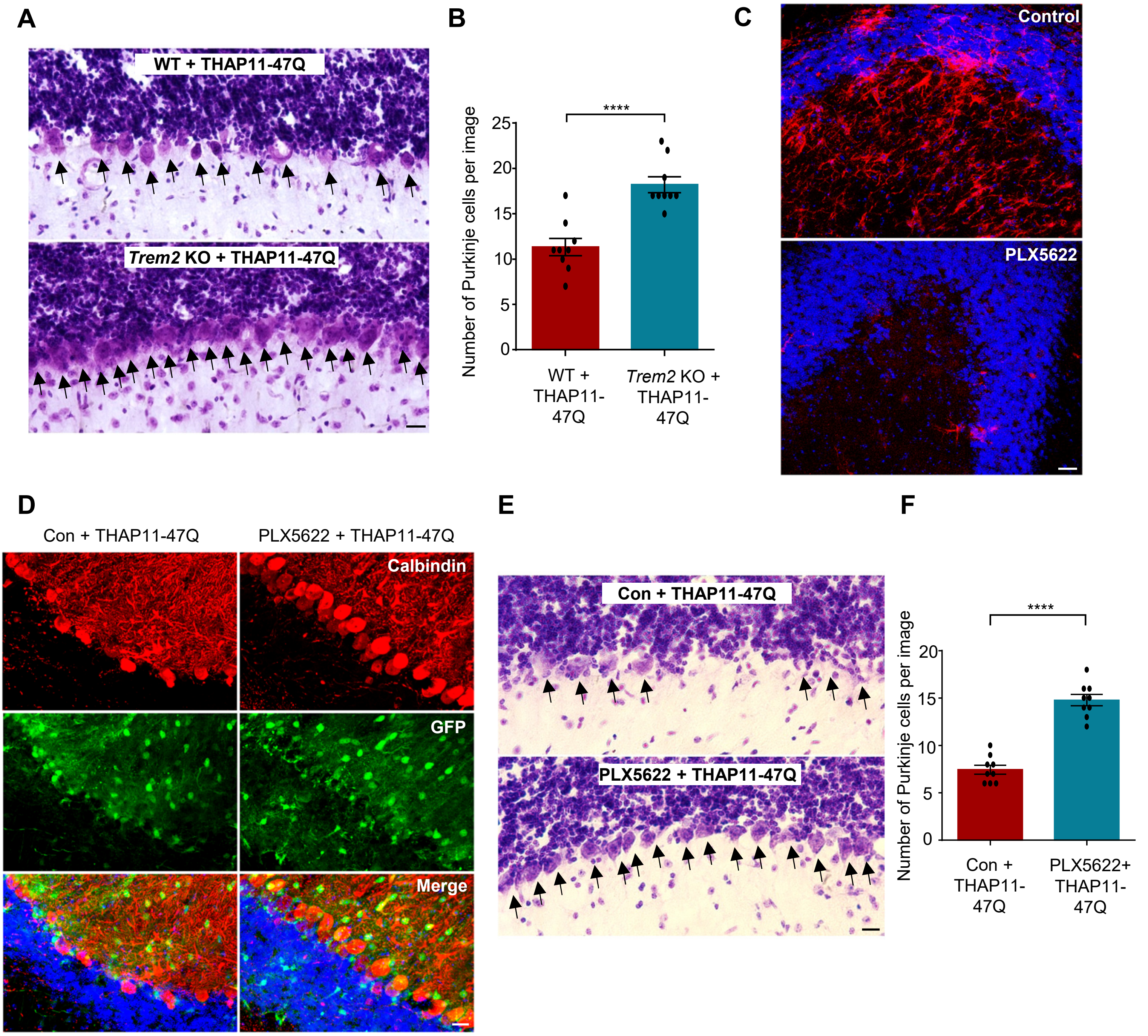

Supplemental Figure 12. Microglial depletion attenuates mutant THAP11 neurotoxicity (related to Figure 8). (A) Nissl staining of cerebellum slices from WT and *Trem2* KO mice injected with AAV-THAP11-47Q (arrows indicate Purkinje cells; scale bar: 20 μm). **(B)** Quantification of Purkinje cell number in Supplemental Figure 12A (n = 9 from three mice, two-tailed student t-test). **(C)** Immunofluorescent staining of IBA1 in the cerebellum of WT mice injected with AAV-THAP11-47Q, treated with control or PLX5622 diet (scale bar: 50 μm). **(D)** Immunofluorescent staining of calbindin in the cerebellum of WT mice injected with AAV-THAP11-47Q, treated with control or PLX5622 diet (scale bar: 20 μm). **(E)** Nissl staining of cerebellum slices from WT mice injected with AAV-THAP11-47Q, treated with control or PLX5622 diet (scale bar: 20 μm). **(F)** Quantification of Purkinje cell number in Supplemental Figure 12E (n = 9 from three mice, two-tailed student t-test). ****P < 0.0001. Data are presented as mean values ± SEM.
