# Supplementary material for: Mutant THAP11 causes cerebellar neurodegeneration and triggers TREM2-mediated microglial activation in mice
Source: J Clin Invest. 2025 Jun 3;135(14):e178349. doi: 10.1172/JCI178349 (PMC12259261; doi:10.1172/JCI178349)

Full unedited blots for Figure 1

Figure 1C

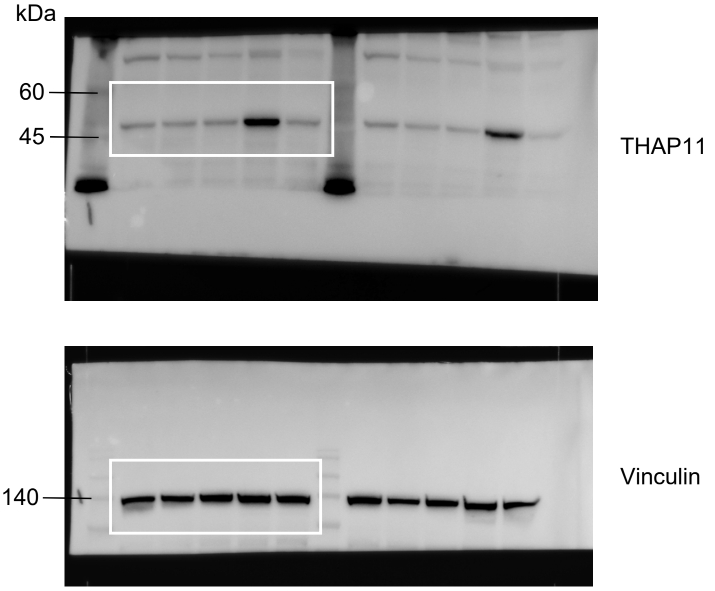

Figure 1F Striatum

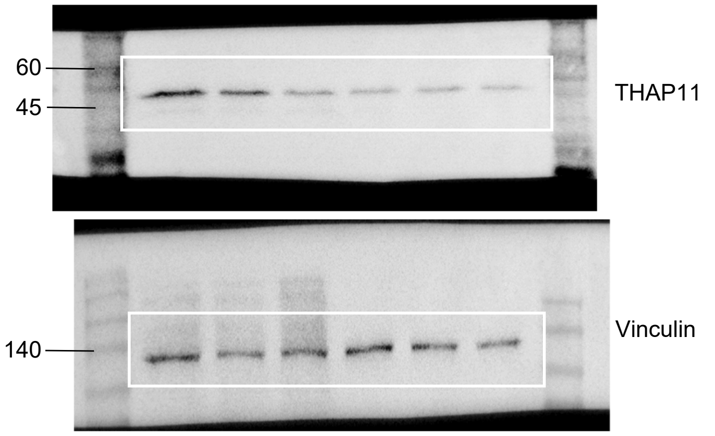

Figure 1F Cerebellum

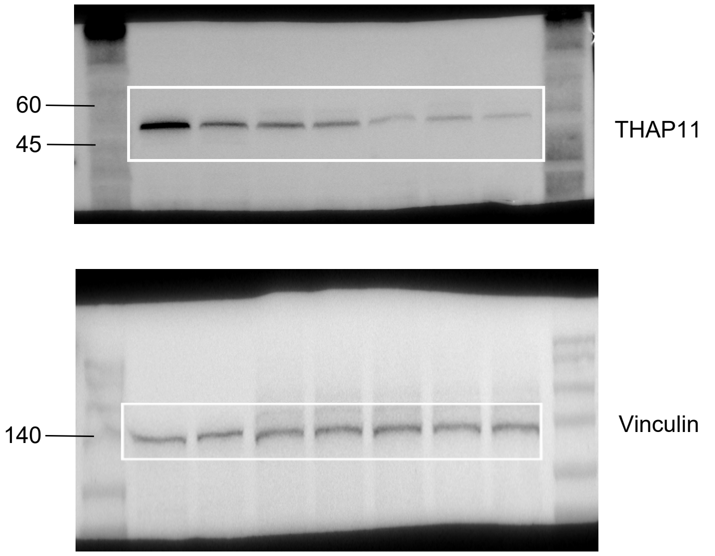

Full unedited blots for Figure 2

Figure 2B Striatum

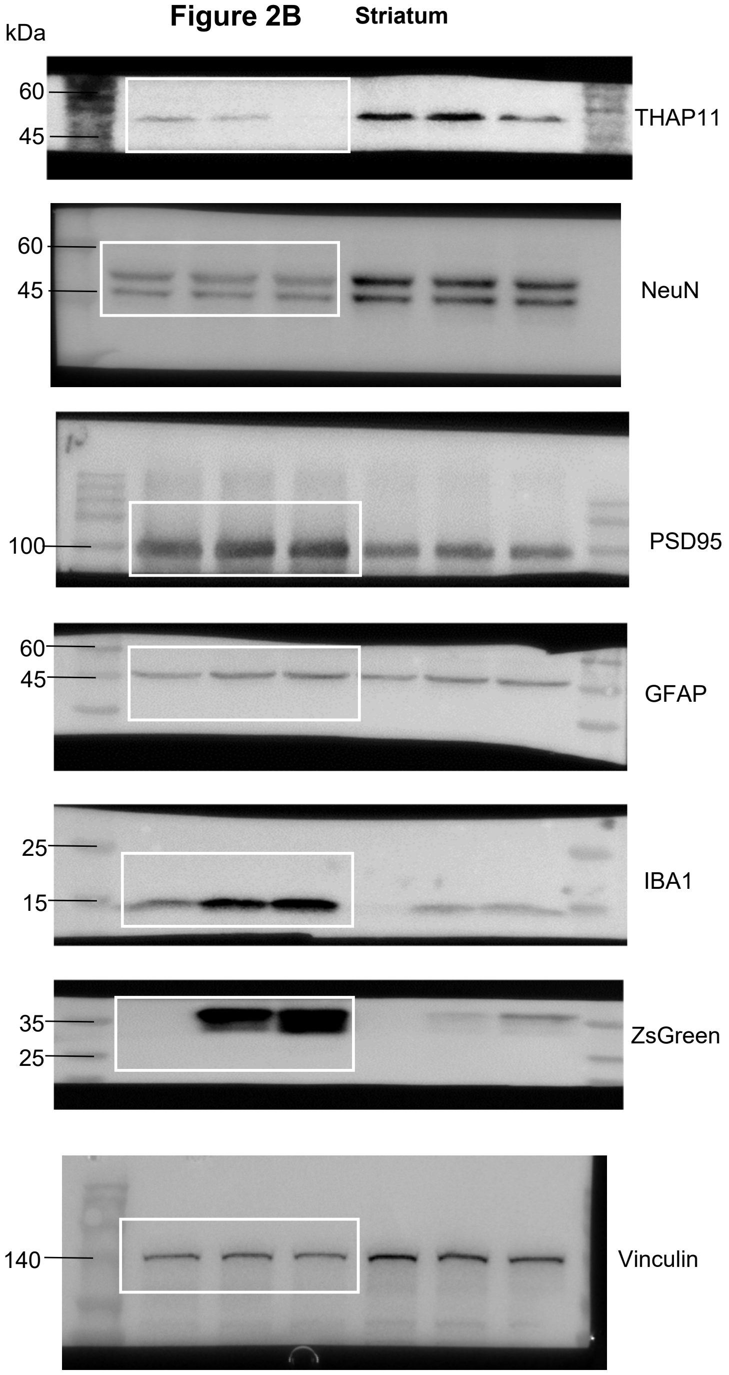

Figure 2B Cerebellum

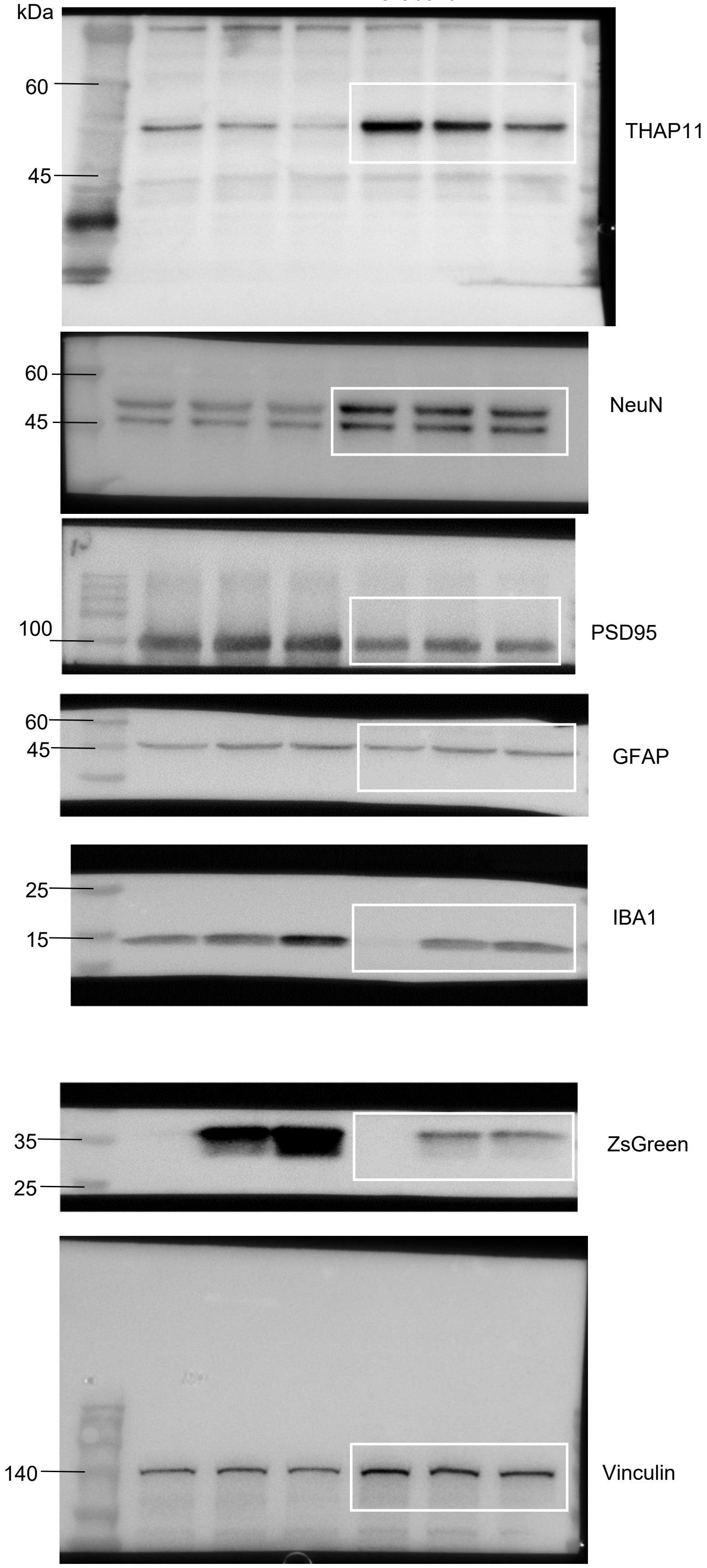

Full unedited blots for Figure 3

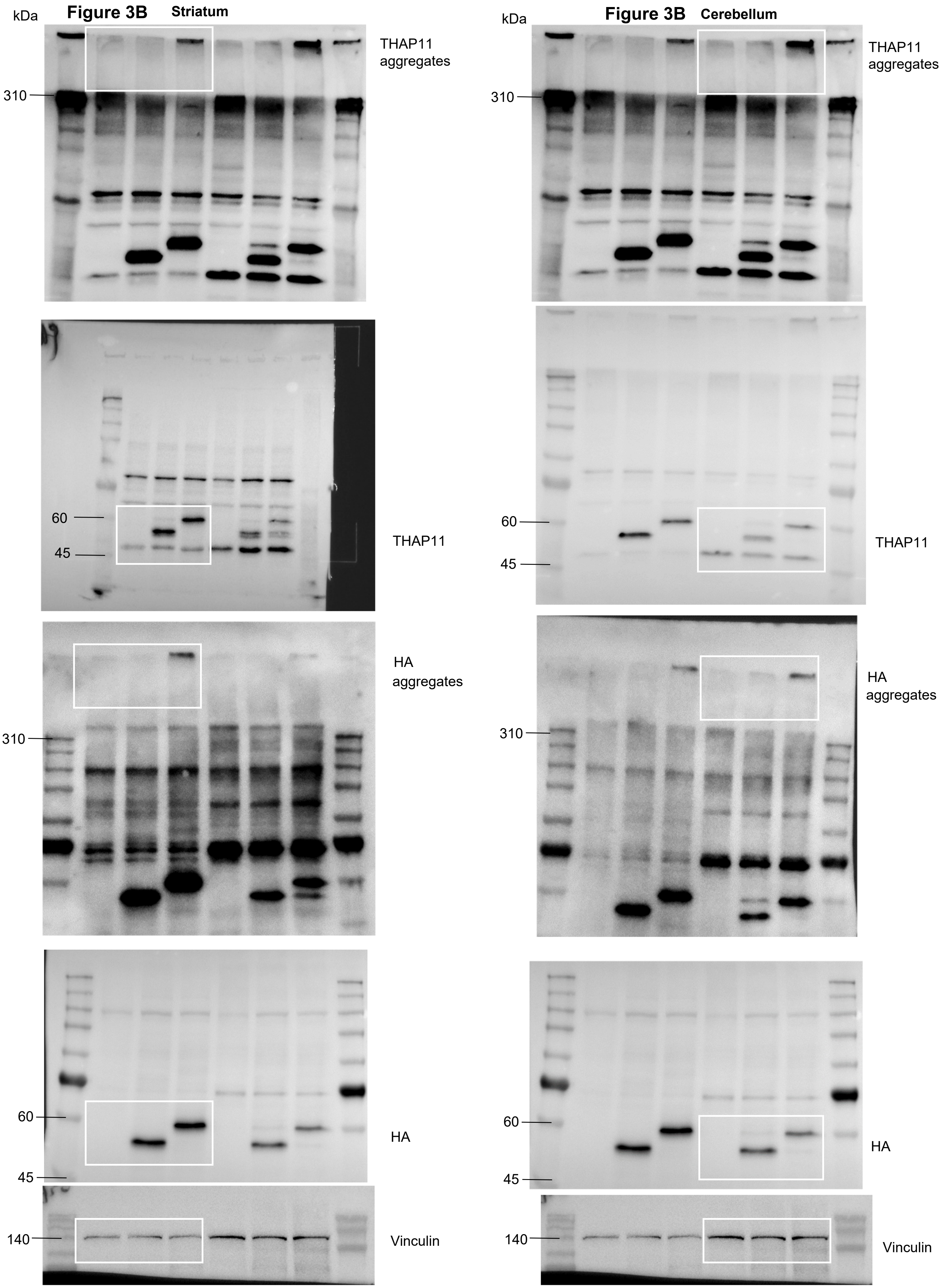

Full unedited blots for Figure 4

Figure 4A Striatum

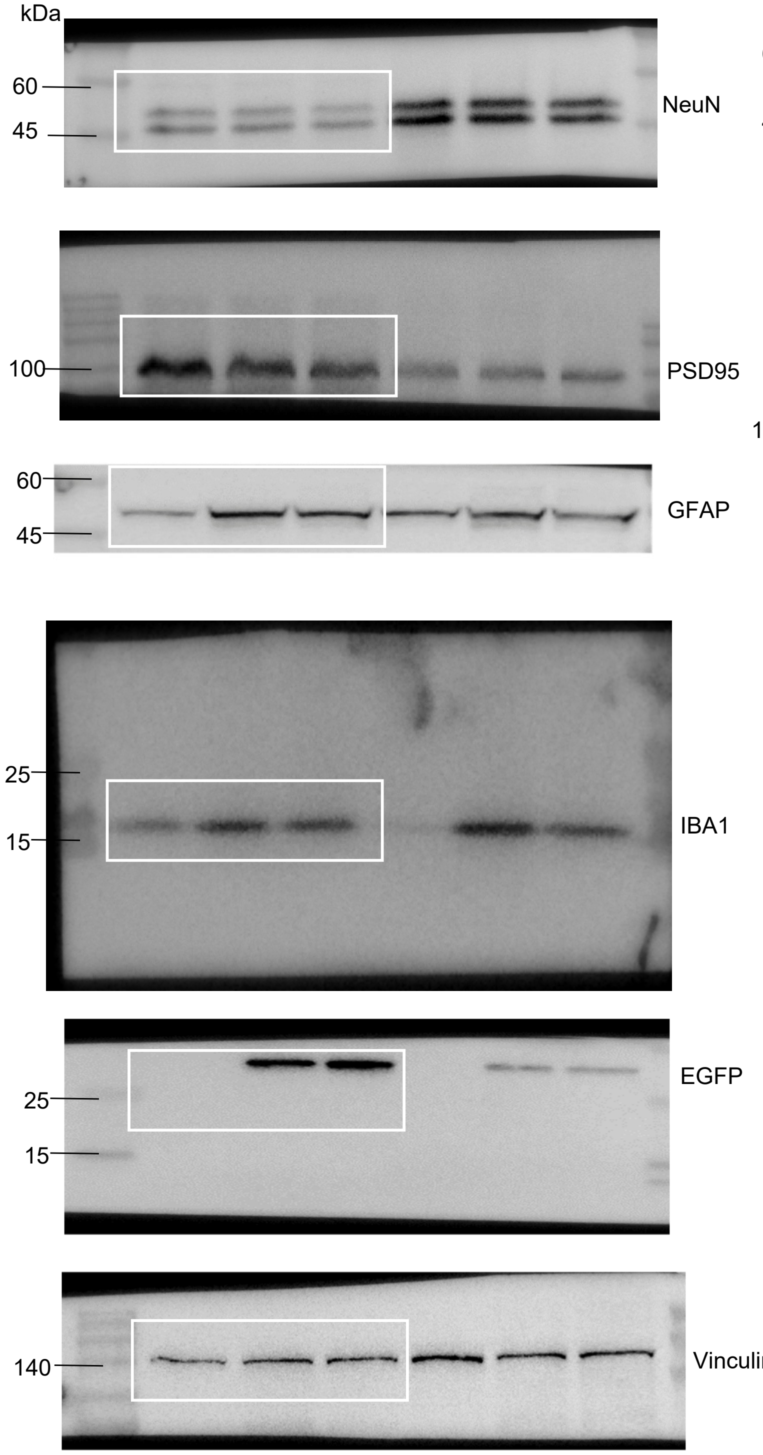

Figure 4A Cerebellum

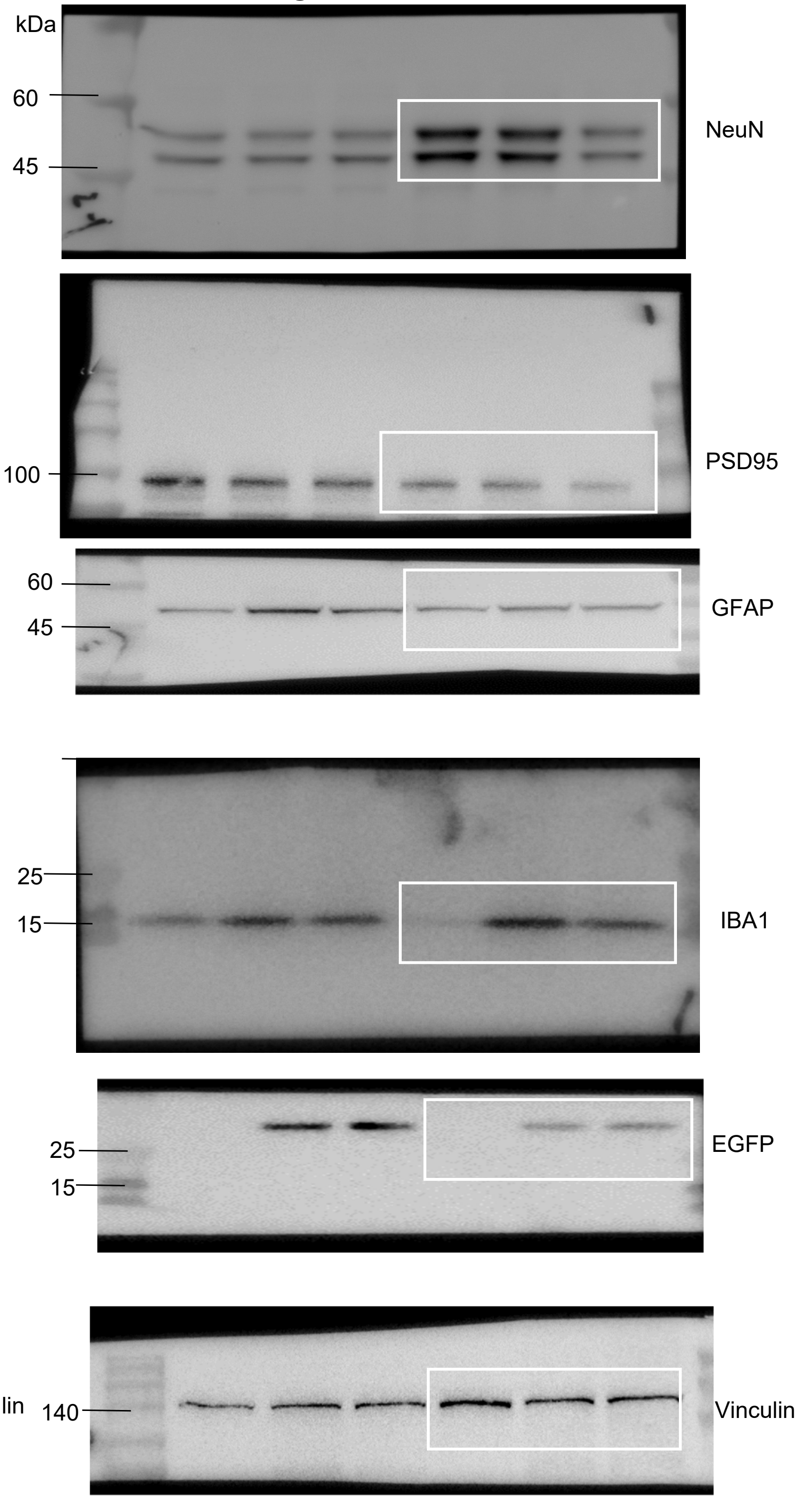

Figure 4C

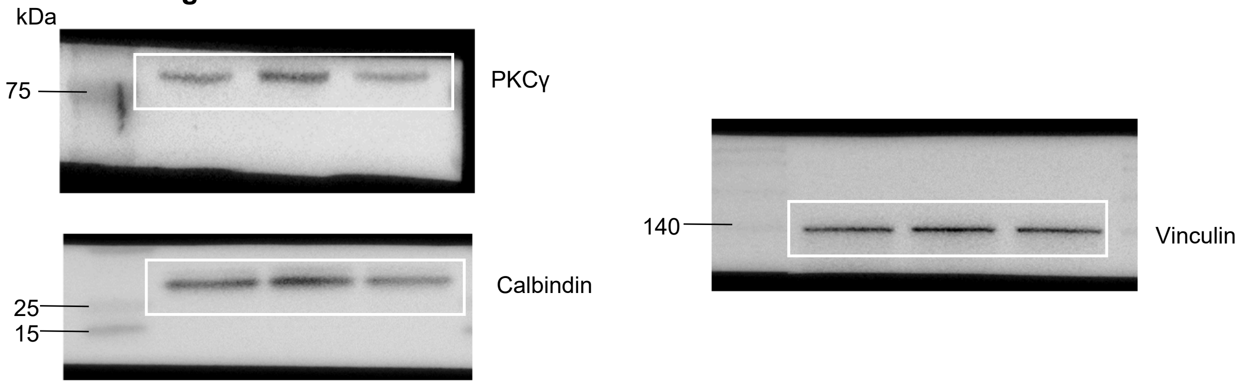

Full unedited blots for Figure 7

Figure 7B

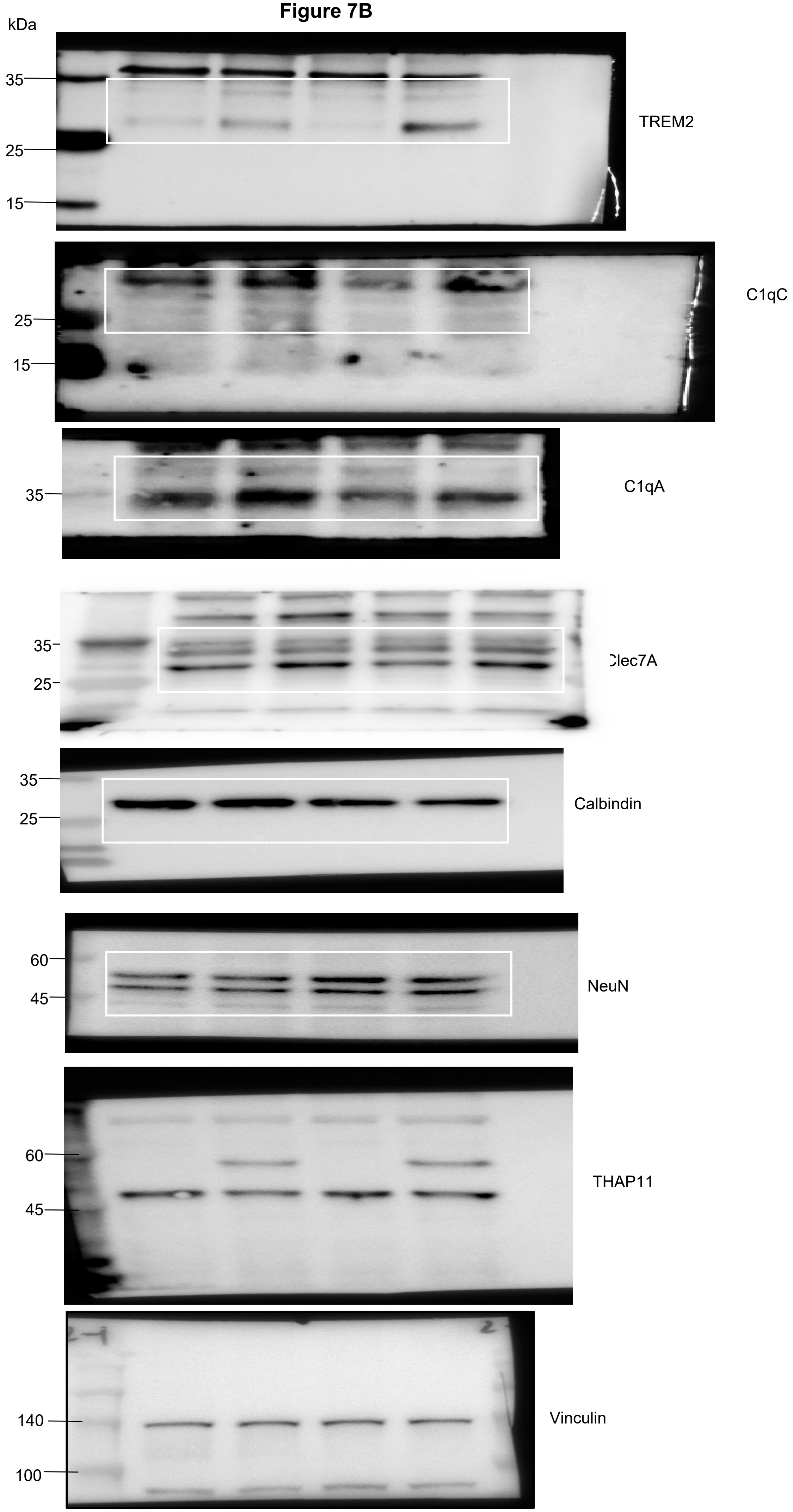

Full unedited blots for Figure 8

Figure 8A

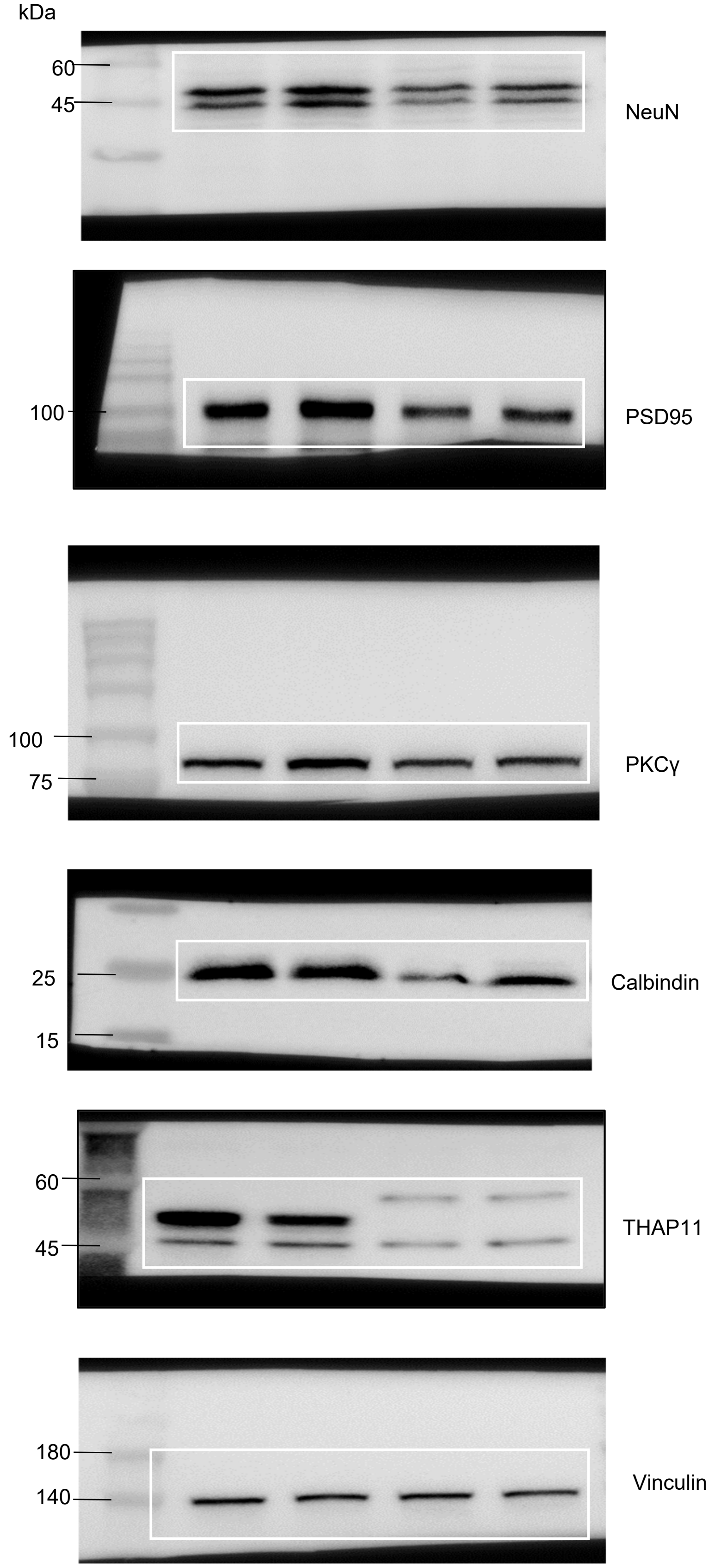

Figure 8E

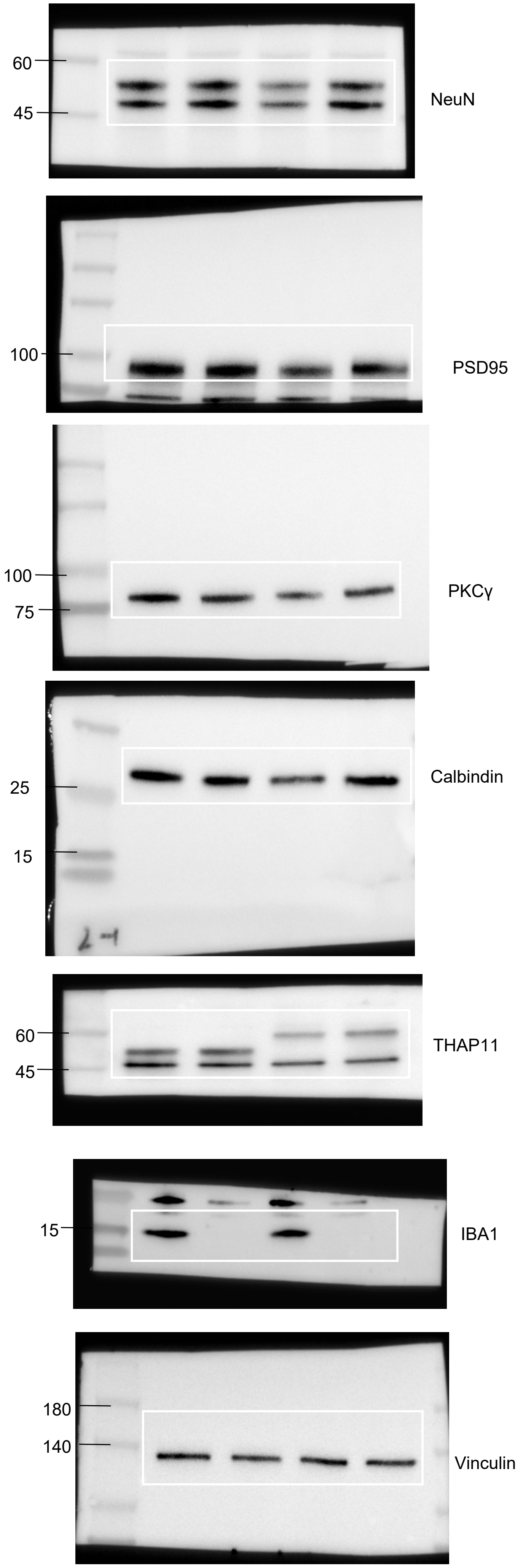

Full unedited blots for Figure S1

Figure S1A

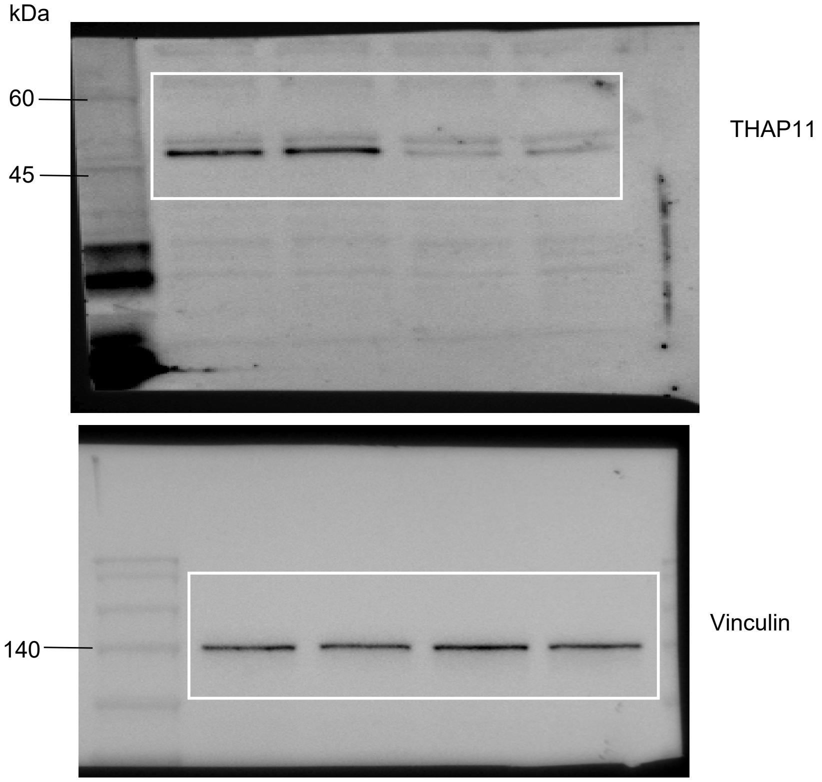

Figure S1B

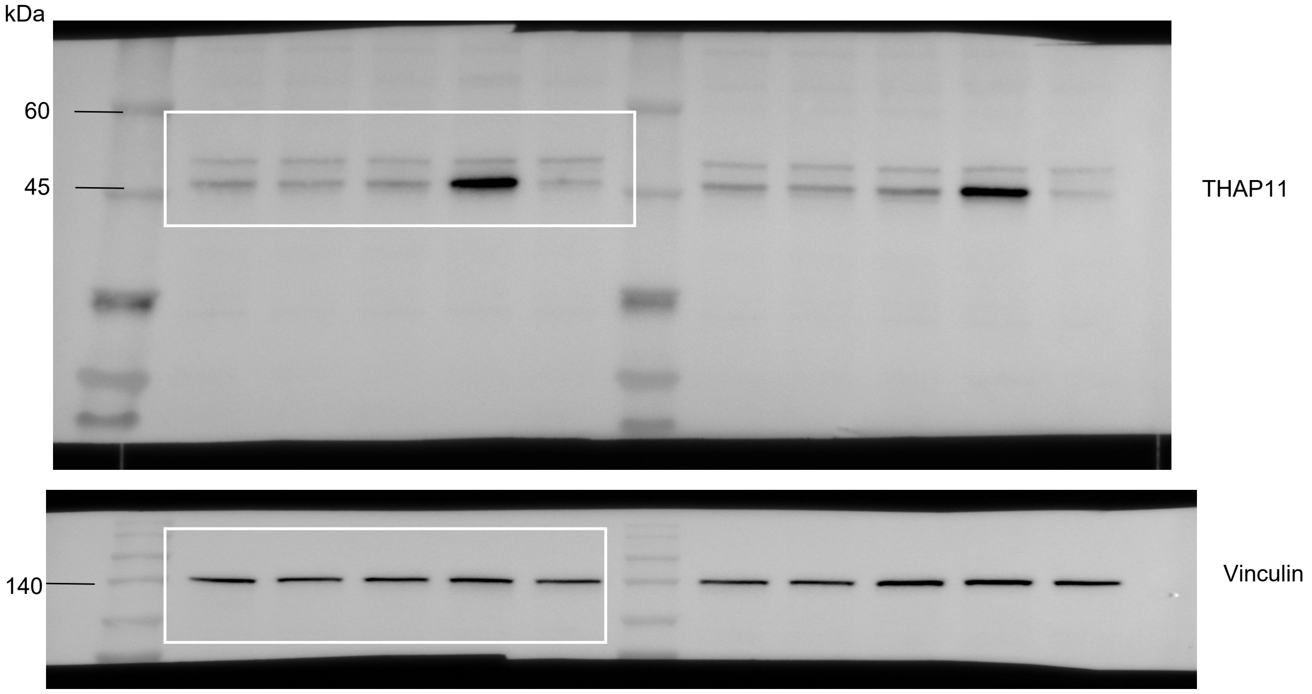

Figure S1D

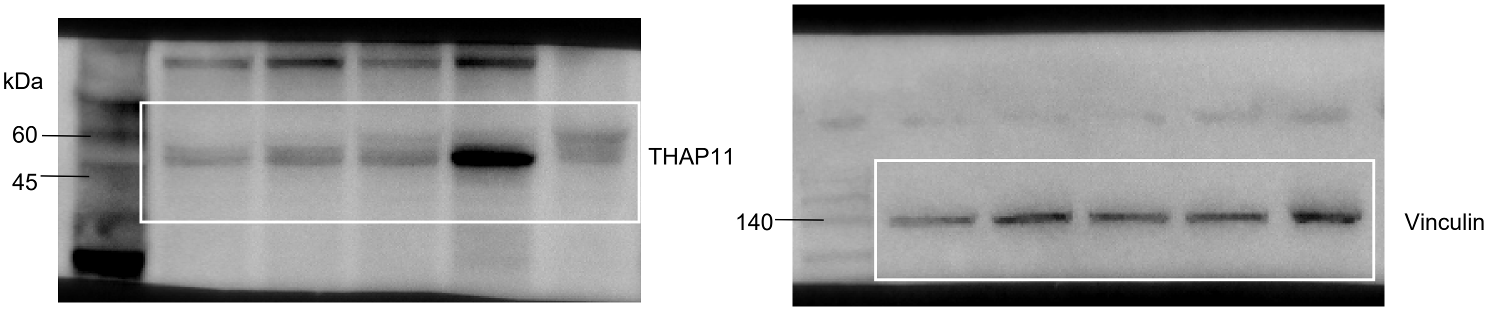

Figure S2B

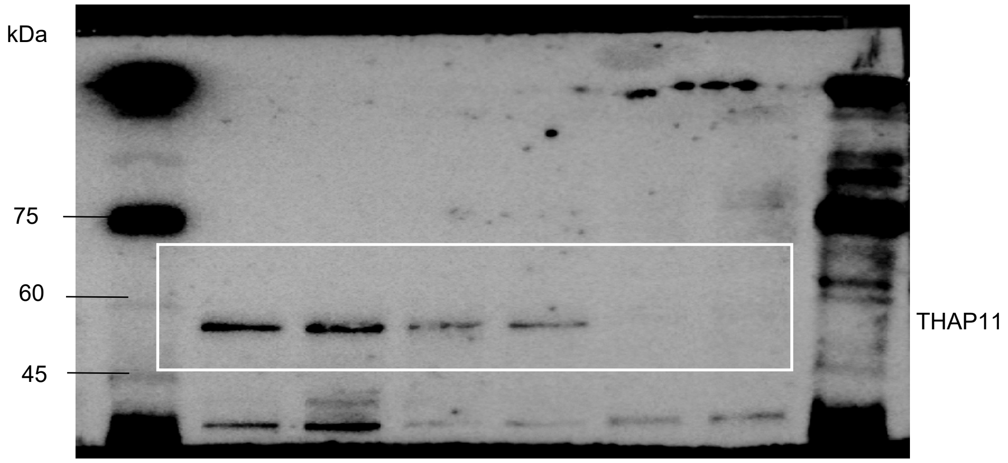

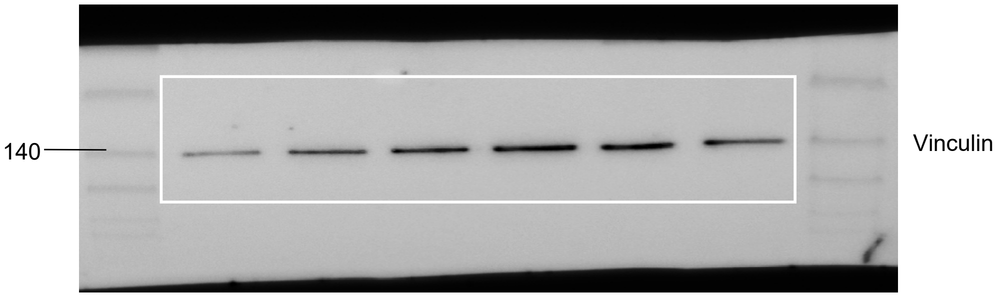

Full unedited blots for Figure S3

Figure S3B

kDa
60
45

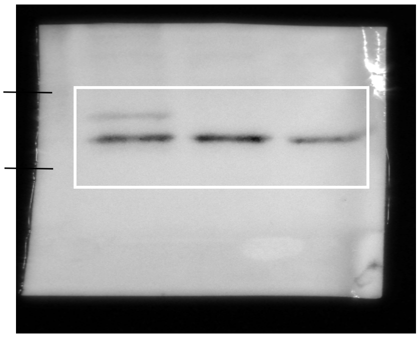

THAP11

180
140

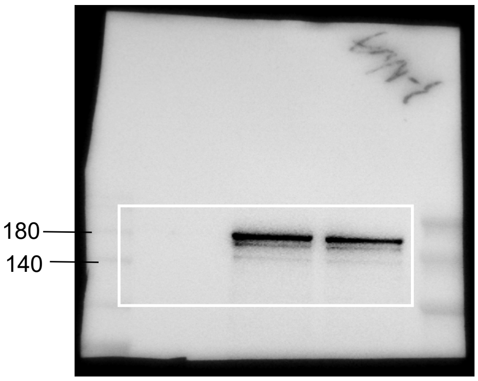

Cas9

35
25

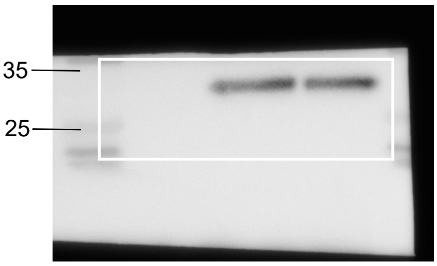

ZsGreen

140

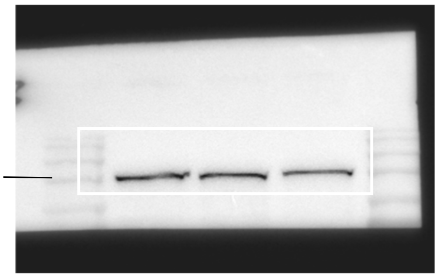

Vinculin

Figure S6A

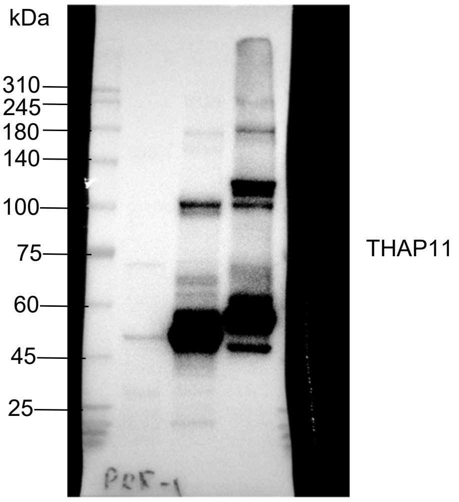
kDa
310
245
180
140
100
75
60
45
25
THAP11
PRK-1

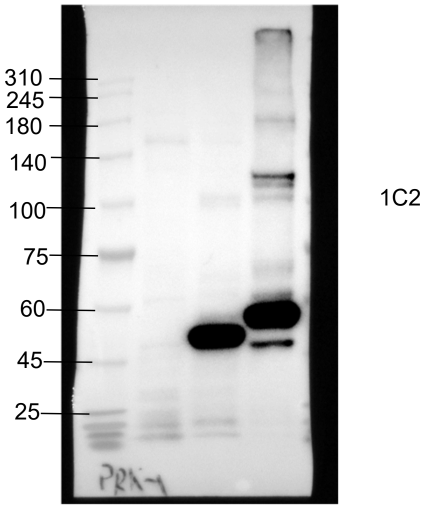
310
245
180
140
100
75
60
45
25
1C2
PRK-1

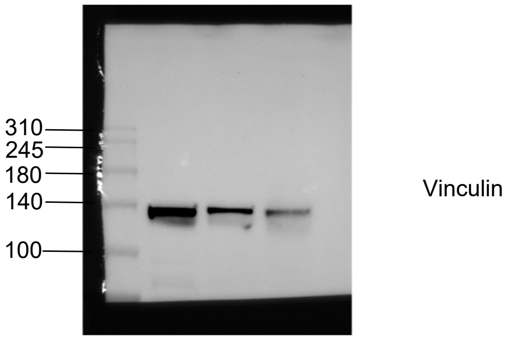
310
245
180
140
100
Vinculin

Full unedited blots for Figure S7

Figure S7A

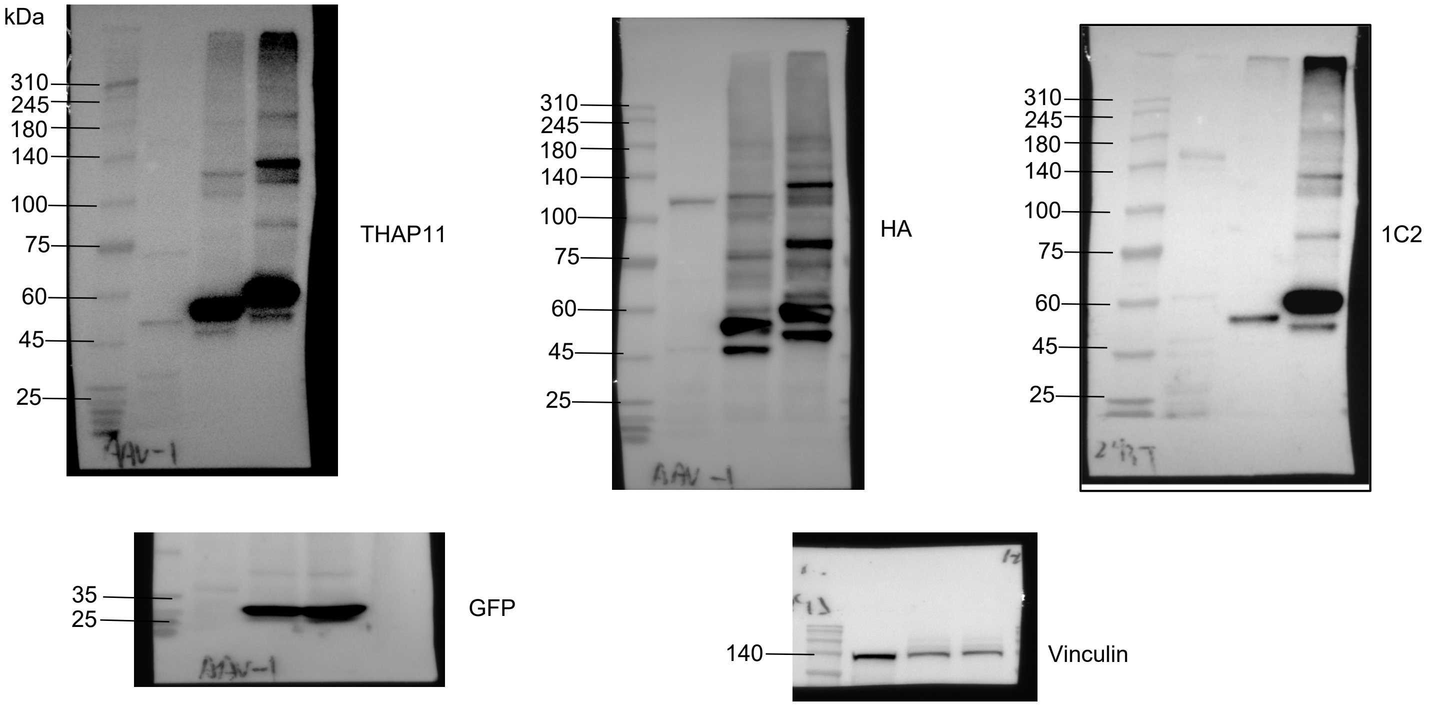

Figure S7E

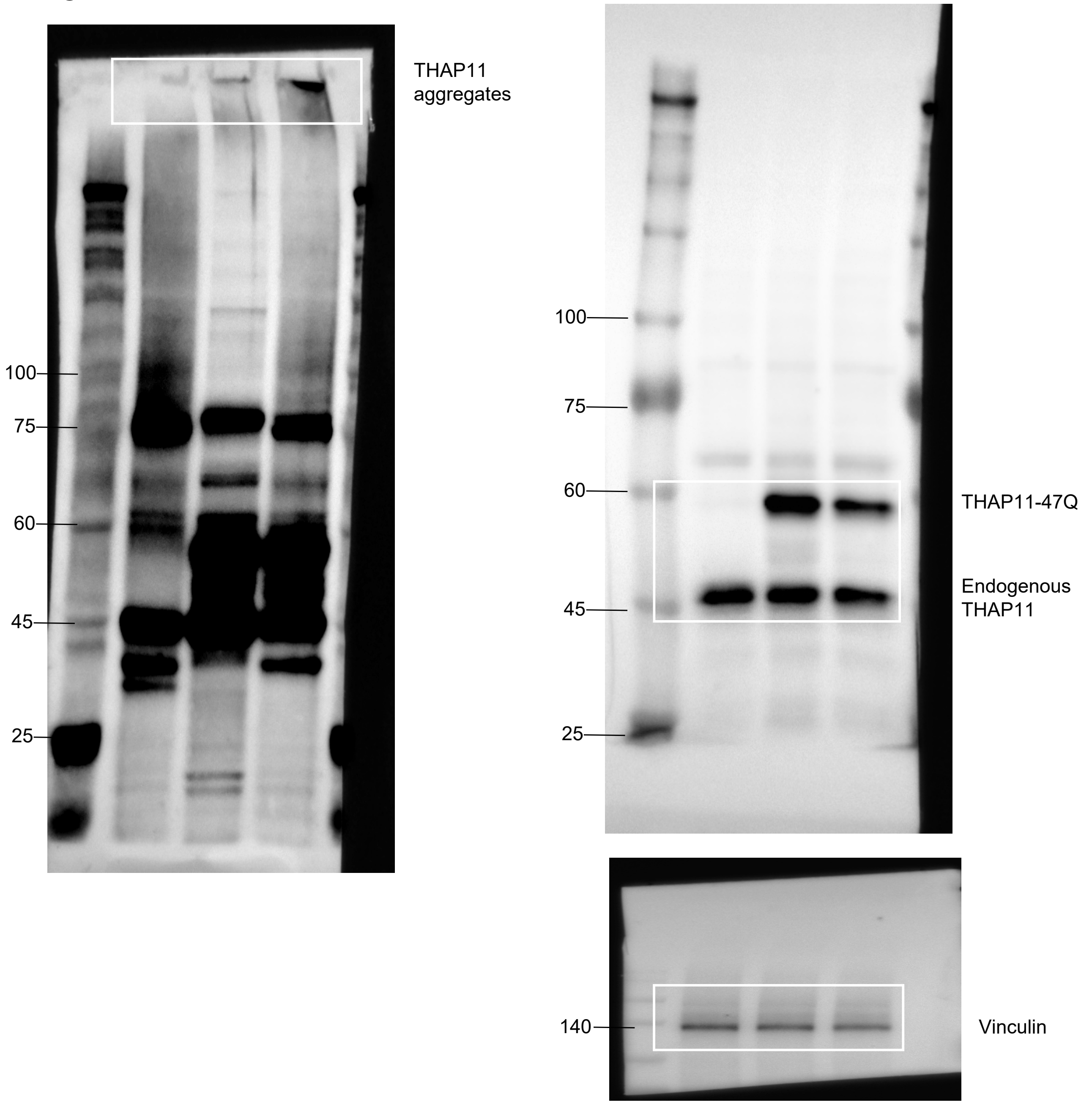

Figure S10A

THAP11

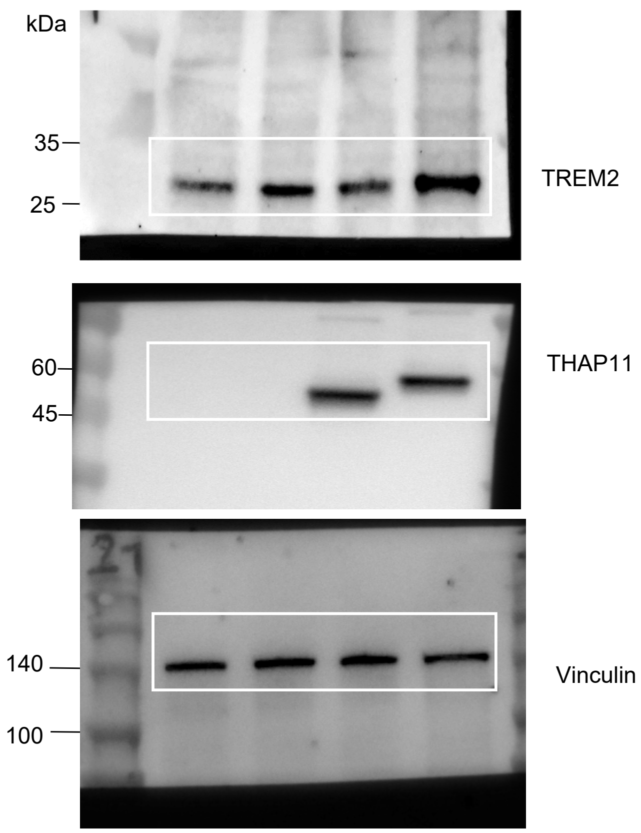

ATXN3

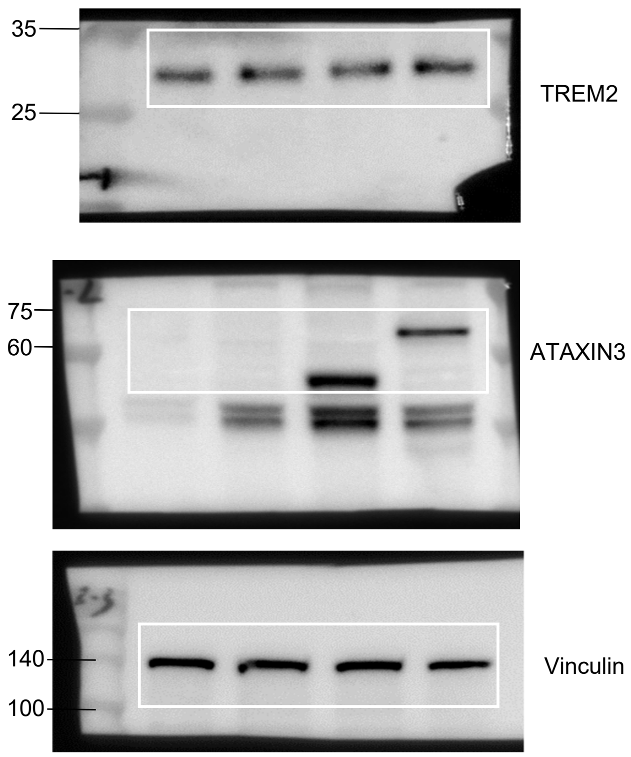

HTT

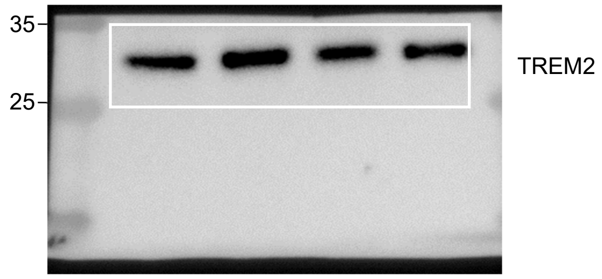

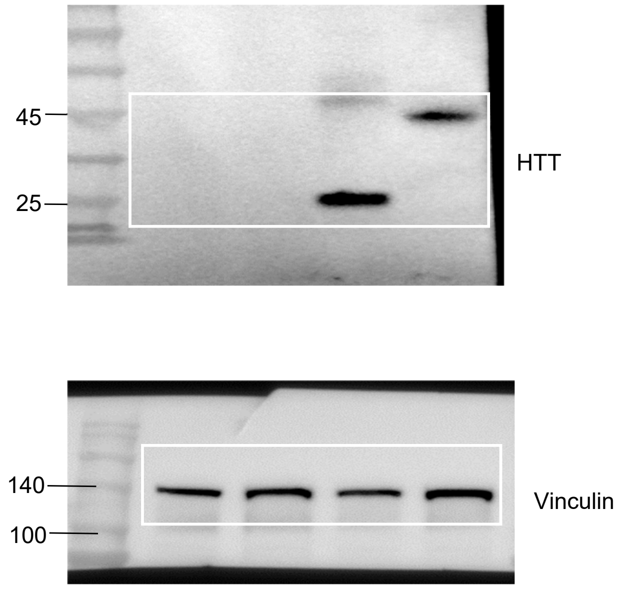

Figure S11E

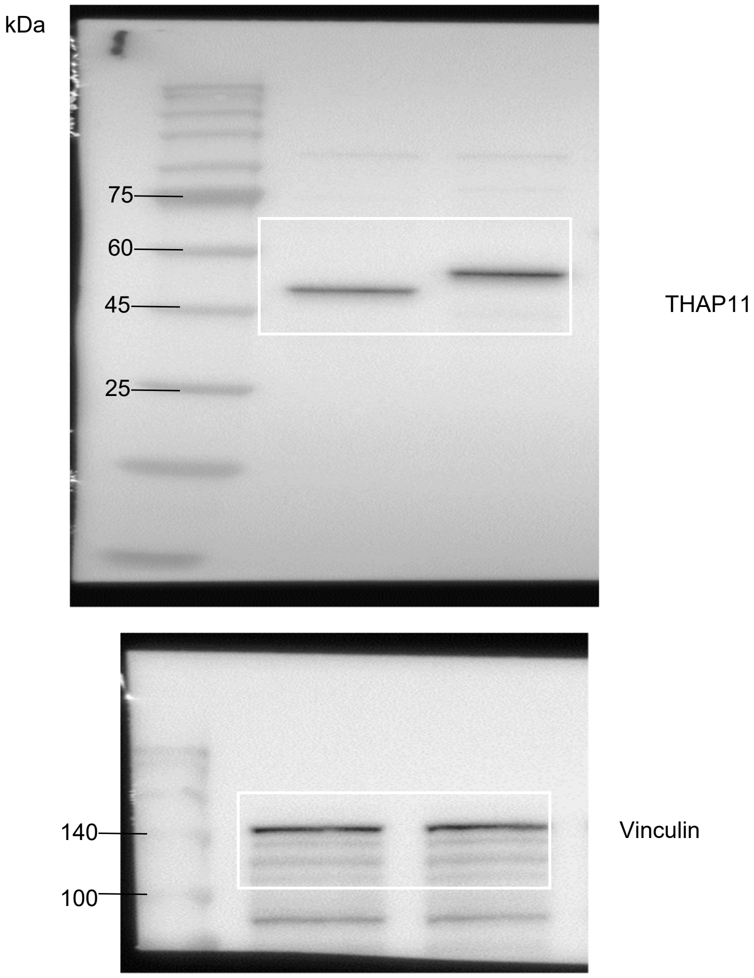

Supplement: Unedited blot and gel images [file jci-135-178349-s181.pdf]
